# High-level chromate resistance in *Arthrobacter *sp. strain FB24 requires previously uncharacterized accessory genes

**DOI:** 10.1186/1471-2180-9-199

**Published:** 2009-09-16

**Authors:** Kristene L Henne, Cindy H Nakatsu, Dorothea K Thompson, Allan E Konopka

**Affiliations:** 1 Department of Biological Sciences, Purdue University, West Lafayette, Indiana 47907, USA; 2 Department of Agronomy, Purdue University, West Lafayette, Indiana 47907, USA; 3 Biological Sciences Division, Pacific Northwest National Laboratory, Richland, WA 99352, USA

## Abstract

**Background:**

The genome of *Arthrobacter *sp. strain FB24 contains a chromate resistance determinant (CRD), consisting of a cluster of 8 genes located on a 10.6 kb fragment of a 96 kb plasmid. The CRD includes *chrA*, which encodes a putative chromate efflux protein, and three genes with amino acid similarities to the amino and carboxy termini of ChrB, a putative regulatory protein. There are also three novel genes that have not been previously associated with chromate resistance in other bacteria; they encode an oxidoreductase (most similar to malate:quinone oxidoreductase), a functionally unknown protein with a WD40 repeat domain and a lipoprotein. To delineate the contribution of the CRD genes to the FB24 chromate [Cr(VI)] response, we evaluated the growth of mutant strains bearing regions of the CRD and transcript expression levels in response to Cr(VI) challenge.

**Results:**

A chromate-sensitive mutant (strain D11) was generated by curing FB24 of its 96-kb plasmid. Elemental analysis indicated that chromate-exposed cells of strain D11 accumulated three times more chromium than strain FB24. Introduction of the CRD into strain D11 conferred chromate resistance comparable to wild-type levels, whereas deletion of specific regions of the CRD led to decreased resistance. Using real-time reverse transcriptase PCR, we show that expression of each gene within the CRD is specifically induced in response to chromate but not by lead, hydrogen peroxide or arsenate. Higher levels of *chrA *expression were achieved when the *chrB *orthologs and the WD40 repeat domain genes were present, suggesting their possible regulatory roles.

**Conclusion:**

Our findings indicate that chromate resistance in *Arthrobacter *sp. strain FB24 is due to chromate efflux through the ChrA transport protein. More importantly, new genes have been identified as having significant roles in chromate resistance. Collectively, the functional predictions of these additional genes suggest the involvement of a signal transduction system in the regulation of chromate efflux and warrants further study.

## Background

*Arthrobacter *species are high G+C Gram positive bacteria that are prevalent in both pristine and polluted soils [1-3]. Although *Arthrobacter *spp. have been noted for their high levels of resistance to a variety of toxic metals [4,5], very little is known about the genetic basis or regulatory mechanisms underlying metal resistance in this genus. *Arthrobacter *sp. FB24 was isolated from soils contaminated with lead-chromate salts and was selected for detailed study based on its high tolerance to a wide assortment of toxic heavy metals [6-8]. Most notably, this strain can survive in the presence of 200 mM potassium chromate in dilute nutrient broth [6]. Reported resistance levels for other *Arthrobacter *species range from 2 to 48 mM chromate [9,10].

The mechanism of chromium resistance in *Arthrobacter *strains remains enigmatic. Although some strains can reduce toxic Cr(VI) to less toxic Cr(III) [11,12], chromate reduction is not typically considered a resistance mechanism [13]. However, chromate efflux has only been biochemically identified as a resistance mechanism in *Proteobacteria *[14-17]. The earliest analyses of efflux-mediated chromate resistance have been performed in *Cupravidus metallidurans *and *Pseudomonas aeruginosa*, and until recently, these two organisms have served as the model organisms for chromate efflux. As a structural analog of sulfate (SO_4_^2-^), chromate enters cells through sulfate uptake systems [18]. Chromate efflux occurs via the ChrA protein in *P. aeruginosa *and *C. metallidurans *and resulted in resistance levels of 4 and 0.3 mM, respectively [19-21]. It is important to note that the number and arrangement of chromate resistance genes differs between these two strains [13,15,20,21]. In addition, in 2007 at least 135 ChrA orthologs were noted in other bacteria as members of the CHR superfamily of chromate transporters [22,23]. There is considerable variation in the genomic context surrounding ChrA orthologs [22], which raises the question as to whether functional or regulatory differences in chromate efflux among organisms bearing ChrA orthologs also exist. Although the CHR superfamily includes representatives from all domains of life, at the time of its construction, the phylogeny was largely dominated by Proteobacteria (35 out of 72 organisms). Moreover, given the high levels of chromate resistance among Actinomycetales such as *Arthrobacter *[2-5], the 135 ChrA orthologs (which includes only three representatives within the order Actinomycetales, *Corynebacterium glutamicum, C. efficiens *and *Kineococcus radiotolerans*) reported by Ramirez-Diaz *et al *[22] is very likely an underestimate of the range of this protein family and warrants further investigation.

Chromate resistance levels reported for bacterial strains with ChrA orthologs are also highly variable, ranging from 0.3 to 200 mM Cr(VI). It is apparent that the mere presence of a *chrA *gene cannot explain this vast difference in resistance levels. Thus, further study of ChrA orthologs and their genomic neighborhoods in a greater diversity of chromate-resistant organisms will undoubtedly yield additional functional and regulatory elements that are relevant to different levels of chromium resistance found in diverse taxa. In this work, we examine such a chromate resistance determinant found in *Arthrobacter *sp. FB24.

## Results

### Identification of a chromate resistance determinant (CRD) in *Arthrobacter *sp. strain FB24

**Figure 1:**
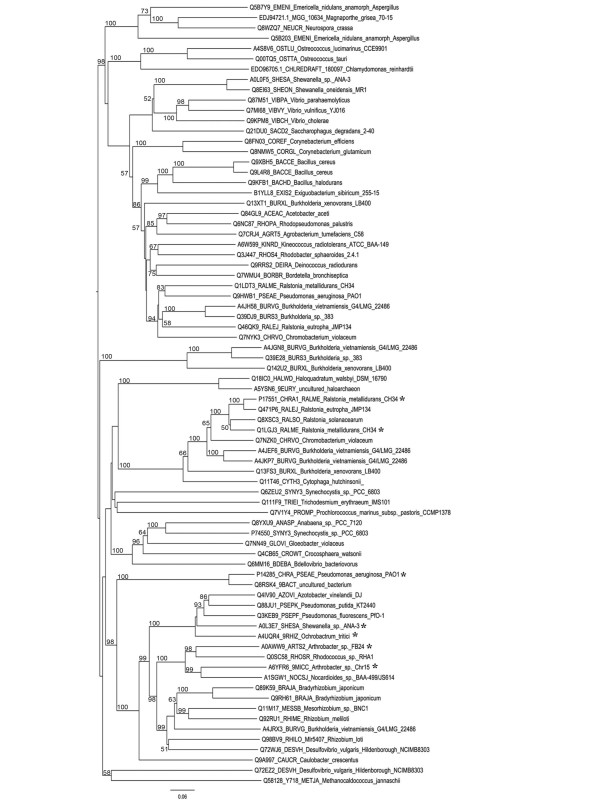
**Phylogenetic Tree of ChrA Orthologs**. Phylogenetic tree of LCHR proteins generated from a subset of the alignment of 513 putative chromate ion transport sequences using ClustalX and default setting for Gonnet series for protein weight matrix (34). Neighbor Joining tree graphically viewed using the FigTree program http://tree.bio.ed.ac.uk/software/figtree/. Branched tips labeled with Uniprot protein accession number, sequence name and species name. Sequences with function supported with experimental data marked with asterisk. Scale bar indicates 0.06 amino acid substitutions per site. Branch ends labeled with bootstrap values >50%. Full tree available in the figure in Additional file 1 and all sequences used are listed in the table provided in Additional file 2.

*Arthrobacter *sp. strain FB24 genome analysis deduced a 450 amino acid (aa) sequence Arth_4248 with similarity to chromate ion transporters. Phylogenetic analysis of the sequence with 512 other characterized and putative ChrA sequences (see Figure 1 and Additional files 1 and 2) suggests that it forms a new branch in the CHR superfamily [22] that is composed of Actinobacteria. This group likely has unique evolutionary features since the majority (70%) of ChrA ortholog sequences used in the comparison is from Proteobacteria yet it formed its own branch. In fact, most of the clades are composed of specific phyla/classes of biota (Additional file 1).

**Figure 2:**
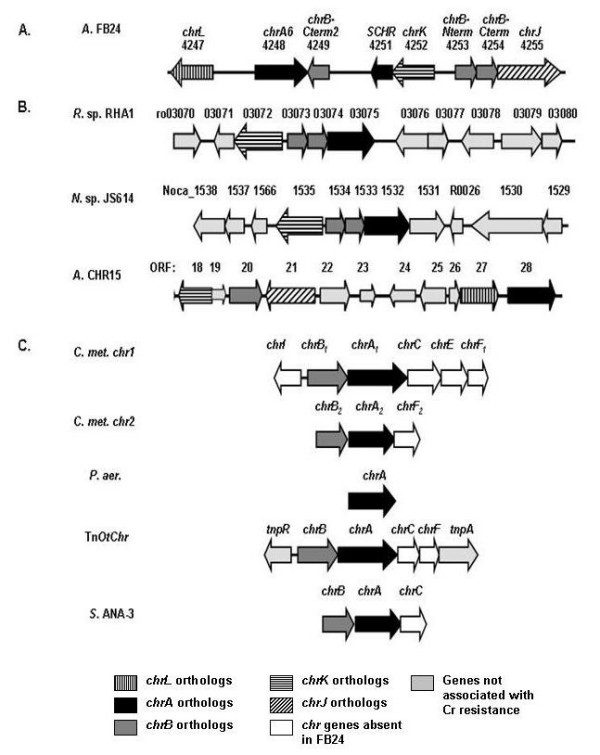
**Comparison of genetic determinants of chromate resistance as studied in other bacterial strains versus *Arthrobacter *sp. strain FB24**. *R*. sp. RHA1, *Rhodococcus *sp. RHA1 [GenBank: NC_008268]; *N*. sp. JS614, *Nocardiodes *sp. JS614 [GenBank: NC_008699]; *A. CHR15*, *Arthrobacter sp*. CHR15 plasmid pCHR15 [6,35]; *C. met. chr1 *and *chr2*, *C. metallidurans *chromate resistance determinants 1 (plasmid pMOL28) and 2 (chromosomal) [21]; *P. aer*., *Pseudomonas aeruginosa *plasmid pUM505 [20]; *TnOtChr*, transposable element from *Ochrobactrum tritici *5bv11 [58]; *S*. ANA-3, *Shewanella *sp. strain *chrBAC *operon, plasmid 1 [GenBank: CP000470]. Drawing not to scale.

The genome neighborhood of Arth_4248 consists of a 10.6-kb region of five putative chromate resistance genes and three proximal genes of unknown function located on a 96-kb plasmid (Figure 2). Of five genes similar to ones associated with Cr(VI) resistance in other organisms, two encode ChrA efflux protein orthologs (Arth_4248 and 4251) and three are similar to different regions of a putative regulatory protein, ChrB (Arth_4249, 4253 and 4254). The remaining three genes (Arth_4247, 4252 and 4255) have not been previously shown to be associated with chromate resistance. The region between Arth_4251 and Arth_4249 is an approximate 1.3 kb region of low complexity. Currently, there is no strong indication of functional genes within this region.

**Figure 3:**
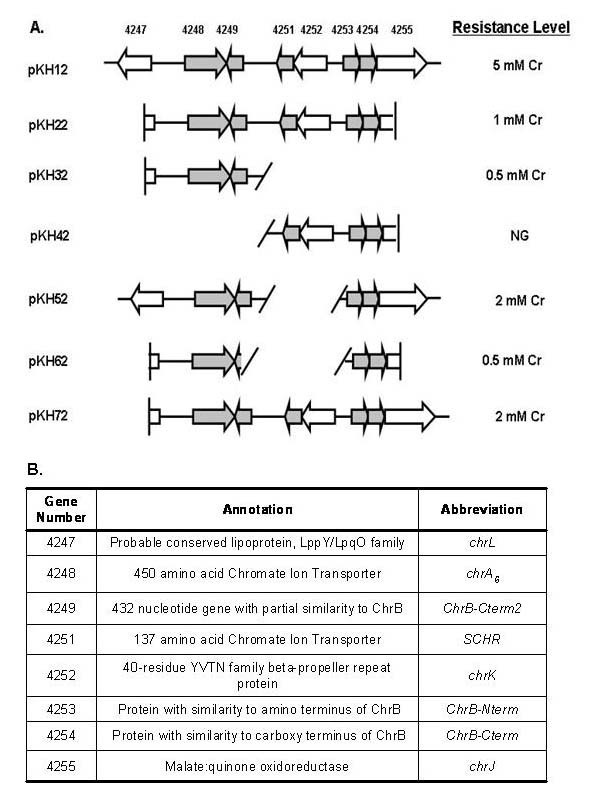
**Schematic of constructs used in complementation experiments with strain D11**. Panel A: 10.6 kb region of FB24 plasmid 3. Numbers correspond to the following genes: Arth_4255 (*chrJ*), Arth_4254 (*ChrB-Cterm*), Arth_4253 (*ChrB-Nterm*), Arth_4252 (*chrK*), Arth_4251 (*SCHR*), Arth_4249 (*ChrB-Cterm2*), Arth_4248 (*ChrA*_6_), Arth_4247 (*chrL*). Genes present in each of the constructs and chromate resistance levels on 0.1X NA plates. NG = No growth. D11 transformed with vector pART2 only did not grow on Cr. Panel B: Designated gene names and corresponding gene numbers used within text.

The chromate resistance determinant in *Arthrobacter *sp. strain FB24 has a similar genetic arrangement to that found in chromate-resistant *Arthrobacter *sp. CHR15, but is markedly different than in the two well-studied Proteobacteria, *P. aeruginosa *and *C. metallidurans *(Figure 2). More recently, a transposable element conferring chromate resistance in *Ochrobactrum tritic *was found to have a similar genetic makeup to the *chr1 *determinant in *C. metallidurans *[17], while a chromate resistance operon containing *chrA, chrB *and *chrC *was found in *Shewanella *sp. strain ANA-3 [16]. Additional genes involved in chromate resistance in *C. metallidurans*, such as the superoxide dismutase gene *chrC, chrI *and *rpoH *[21] are not present within the CRD of strain FB24. This could point to functional and regulatory differences in chromate resistance between these distantly related taxa. Thus, we were led to investigate Arth_4247, 4252 and 4255, as well as previously characterized *chrA *and *chrB *sequences. Due to the potential involvement of Arth_4247, 4252 and 4255 in chromate resistance, we have named these genes *chrL, chrK and chrJ*, respectively (Figures 2 and 3).

### Sequence analysis of the CRD

Arth_4248, the putative 450 amino acid (aa) chromate ion transporter, is most similar to ChrA from *Rhodococcus *sp. RHA1 (79%). The protein is predicted to have 12 transmembrane helices and two CHR domains defined by a conserved GGX_12_VX_4_WX_16_PGPX_10_GX_7_G motif, placing it within the LCHR family of chromate ion transporters [22,24]. However, there is little sequence similarity (35% similarity across 106 of 225 amino acids) between the amino and carboxy halves of Arth_4248; hence, it does not appear to have arisen by direct tandem duplication of the same CHR domain-containing open reading frame. Because of the topological diversity of the CHR superfamily proteins [22,25] and the observed preponderance of conserved residues in the N-terminal half of the *P. aeruginosa *ChrA protein [26], it is expected that the amino and carboxy termini may carry out different functional roles in chromate efflux. Alignment of the amino acid sequence of Arth_4248 with that of *P. aeruginosa *ChrA indicated that residues which resulted in Cr(VI) sensitivity following mutation in *P. aeruginosa *[26] are also conserved in Arth_4248.

The other *chrA *ortholog Arth_4251 is predicted to be 137-aa protein with sequence similarity to known ChrA transporters. The protein sequence aligns to the N-terminus of *C. metallidurans *ChrA1 with 71% similarity across 49 amino acids. In comparison, Arth_4251 is only 52% similar to the N-terminus of Arth_4248 across 44 amino acids. The CHR domain in Arth_4251 contains the GGX_12_VX_4_W motif, but lacks the PGPX_10_GX_7_G motif. In addition, no definitive transmembrane helices were predicted for Arth_4251. Small (<200 aa) proteins containing a single CHR domain have been recognized as a separate group of proteins within the CHR family. Recently, *Bacillus subtilis *SCHR orthologs *ywrA *and *ywrB *were shown to confer chromate resistance in *E. coli*; however, both genes were required for the resistant phenotype [27]. Genes encoding SCHR proteins are usually present as pairs within a genome [22]. In FB24, though, there does not appear to be a partner SCHR gene for Arth_4251 and the aa sequence is more closely related to CHR domains from LCHR proteins than to those of the SCHR family [23].

Three open reading frames (ORFs) designated Arth_4253, Arth_4254 and Arth_4249 in the putative CRD region share sequence similarity to the *C. metallidurans *ChrB proteins. Arth_4253, which encodes a 171 aa protein, aligns with the N-terminal portion of the *C. metallidurans *ChrB1 protein [GenPept: YP_582012] (42% similarity across 133 aa). Arth_4254 is a predicted 143 aa protein that exhibits 53% similarity across 132 aa of the C-terminal portion of the *C. metallidurans *ChrB1 protein. Together, Arth_4253 and Arth_4254 appear to encode the complete sequence for a full-length ChrB gene, but the gene sequences overlap by 4 nucleotides and a potential Shine-Dalgarno sequence is present upstream of the predicted start codon of Arth_4254. Repeated sequencing of this region did not reveal any potential sequencing errors that could explain this observation. RT-PCR analysis revealed that Arth_4253 and Arth_4254 can form a dicistronic mRNA (operon structure analysis provided in Additional file 3). Arth_4249 contains 430 nucleotides, but does not yield any hits to known genes at the nucleotide level. A BLASTx search of the translated nucleotide sequence versus the protein database shows that the predicted amino acid sequence is 76% similar to Arth_4254 across 77 aa.

Arth_4252 encodes a 344 aa protein containing a 40-residue YVTN family beta-propeller repeat and a WD40 repeat domain (with 81% sequence similarity to ORF18 in *Arthrobacter *sp. strain CHR15) with an N-terminal signal sequence. The function of Arth_4252 is presently unknown, but other proteins within the WD40 repeat domain family are associated with the regulation of signal transduction and sensing membrane stress [28,29]. Arth_4252 also shares 62% sequence similarity to Rmet_6194, which is located approximately 4 kb downstream of the *C. metallidurans chrA1 *gene, Rmet_6202. However, a functional role for Rmet_6194 in chromate resistance in this organism has not been established. Orthologs of Arth_4252 were also found in close proximity to *chrA *genes in *Arthrobacter *sp. strain CHR15 and several species of *Burkholderia *as revealed by a gene ortholog neighborhood search in the Integrated Microbial Genomes database http://img.jgi.doe.gov.

Arth_4247 has an expected protein sequence of 337 aa with a putative overlapping signal sequence and transmembrane helix at the N-terminus, which suggests that it is a membrane-anchored protein. The protein sequence shares 75% aa similarity with lipoproteins of the LppY/LpqO family, which were first described in *Mycobacterium tuberculosis *but have not been functionally characterized. Other mycobacterial lipoproteins have been shown to perform such diverse roles as binding solutes in ABC transporter complexes, sensing environmental stressors and participating in signal transduction mechanisms [30]. *M. tuberculosis*, like strain FB24, is a high GC% Gram positive bacterium of the order Actinomycetales. The role of lipoproteins in the response to Cr(VI) has not been established in other organisms. Other lipoproteins have been shown to participate in the response to divalent metals such as copper and lead [31,32]. In the case of copper, NlpE stimulated the CpxAR envelope stress response pathway in copper-exposed *E. coli *cells [32]; however, it is not known if an analogous function exists for LppY/LpqO proteins. As in the case for Arth_4252, orthologs of Arth_4247 are also present near *chrA *orthologs in *Arthrobacter *sp. strain CHR15 (81% similarity to ORF 27) and *C. metallidurans *(52% similarity to Rmet_6195).

Arth_4255 encodes a putative malate:quinone oxidoreductase of 517 aa with 77% similarity to *Arthrobacter aurescens *TC1 Mqo. This class of proteins generally functions in energy production, but the biochemical role of Arth_4255 in the context of Cr(VI) resistance is not known. In *Agrobacterium tumefaciens*, insertional inactivation of an operon specifying NADH:quinone oxidoreductases similar to malate:quinone oxidoreductases (MrpA, MrpC and MrpD) resulted in the loss of arsenite oxidation. The phenotype was recovered via complementation with the intact Mrp operon [33]. In other bacteria, NADH-dependent oxidoreductases have been shown to reduce Cr(VI) [34]; however, there is no conclusive evidence of Cr(VI) reduction in FB24, and it is unlikely that Arth_4255 is a Cr(VI) reductase.

### Loss of plasmid DNA from strain FB24 results in metal sensitivity and increased intracellular chromium accumulation

A chromate-sensitive mutant (D11) was obtained after successive culturing of FB24 for 90 generations in the absence of chromate. Loss of plasmid DNA was assessed by Southern hybridization using a 10.6-kb probe for the CRD, and the results were validated by a PCR screen using gene-specific primers (data not shown). Strain D11 was hypersensitive to low levels (0.5 mM), whereas the wild type grew prolifically on 0.1X nutrient agar (NA) plates amended with 5 mM chromate. Strain D11 was also very sensitive to lead, zinc and cadmium. Jerke *et al *(2008) had shown that FB24 contained 3 plasmids, each with genes that confer resistance to lead, zinc and cadmium [35]. Whereas FB24 attained maximal cell densities in 200 μM lead, zinc and cadmium in mXBM, growth of strain D11 was strongly inhibited by 10 μM lead, 50 μM zinc and 1 μM cadmium (data not shown).

Total intracellular chromium content was measured in chromate-exposed cells of FB24 and D11 to determine if the loss of chromate resistance in strain D11 correlated with increased intracellular accumulation of chromium. There was a significant difference (*p *= 0.015) in chromium content between strain D11 (2.8 × 10^-7 ^mol mg protein^-1^) and FB24 (9.2 × 10^-8 ^mol mg protein^-1^). Chromium was undetectable in FB24 and D11 cells that were not exposed to chromate. Similar decreases in chromium accumulation were found between chromate-resistant and -sensitive strains of *P. aeruginosa *and *C. metallidurans *which contain ChrA efflux pumps [15,36]. The comparable change in chromium accumulation between resistant and sensitive strains of *Arthrobacter *sp. FB24 and the two organisms in which the operation of an efflux pump has been biochemically demonstrated supports the hypothesis that plasmid-encoded Cr(VI) resistance in strain FB24 results from the function of a ChrA-like efflux pump.

### Complementation of strain D11 with CRD

To localize the essential determinants for chromate resistance within the CRD, a series of plasmids were designed and tested for their capacity to confer chromate resistance in the chromate-sensitive strain D11 (Figure 3). Only D11 transformed with pKH12 (the complete 10.6 kb region) was able to grow comparably to FB24 on 0.1X (NA) plates containing 5 mM chromate (Figure 3). The other transformants, in which regions of the CRD were deleted, were able to grow only at lower levels of chromate (0.5 to 2 mM). In particular, *chrA *produced a resistance level of 0.5 mM Cr(VI) regardless of the presence of *chrB-Nterm *and *chrB-Cterm*.

### Expression of chromate resistance genes in strain FB24 under chromate stress

Quantitative RT-PCR was employed to determine if expression of the chromate resistance genes was inducible by and specific to Cr(VI). Transcription from each of the eight genes of the CRD was induced by increasing concentrations of chromate (Table 1). Five μM chromate was sufficient to detect enhanced expression of each gene. For most genes in the CRD, maximal expression was achieved at 0.1 mM Cr(VI). In the case of *chrB-Nterm*, Arth_4253, maximum transcript abundance occurred at 5 μM chromate and was maintained up to 20 mM Cr(VI). *ChrB-Cterm*2, Arth_4249, exhibited low (2-fold) induction at 5, 25 and 50 μM Cr, followed by a sharp increase in transcript levels at 0.1 mM Cr(VI). Specificity of induction of the CRD genes was assessed with lead, arsenate and hydrogen peroxide, all of which induced little or no expression (Table 2).

**Table 1:** Expression of CRD genes under various levels of chromate stress^a^.

CRD Gene	Basal Expression In 0 mM Cr(VI)^b ^× 10^2^	**Relative Fold Difference**^c^ Cr(VI)/0 mM Cr(VI)						
		
		0.005	0.025	0.05	0.1	5	20	100
***chrL***	4.20 (0.45)	36.7* (9.3)	**95.2** **(8.7)**	69.8 (12.1)	95.1 (42.9)	63.4 (29.7)	45.1* (14.3)	15.3* (3.5)
***chrA***_6_	2.25 (0.36)	8.5* (1.3)	16.2* (3.9)	27.4* (2.5)	**42.1** **(4.2)**	50.7 (14.5)	37.6 (9.8)	22.9 (8.2)
***chrB-Cterm2***	15.6 (4.95)	2.0* (0.3)	2.2* (0.5)	2.5* (0.5)	**7.1** **(2.6**)	6.3 (1.8)	8.0 (3.2)	2.0* (0.8)
***SCHR***	8.50 (2.06)	1.9* (0.5)	4.7* (0.6)	5.1* (0.7)	**7.8** **(0.7)**	6.8 (1.9)	5.1 (1.2)	2.1* (0.9)
***chrK***	21.9 (2.89)	3.7* (0.5)	6.1* (0.7)	**7.5** **(1.9)**	10.1 (1.9)	7.2 (1.6)	6.9 (1.6)	4.4 (1.4)
***chrB-Nterm***	249 (86.4)	**8.0** **(2.6)**	12.5 (4.0)	13.8 (5.6)	18.0 (8.0)	16.9 (7.1)	14.0 (6.5)	4.2 (1.5)
***chrB-Cterm***	0.51 (0.04)	4.3* (0.7)	8.4* (2.1)	16.0* (1.5)	**21.3** **(2.0)**	25.4 (4.4)	30.9 (6.0)	15.3 (5.5)
***chrJ***	1.23 (0.40)	7.2* (1.5)	14.3* (2.8)	19.0* (2.5)	**37.0** **(15.0)**	92.4 (47.2)	47.6 (13.2)	19.2 (6.7)

^a ^The basal (0 mM Cr(VI)) transcript levels are given in copy number/ng total RNA. For the remaining concentrations of Cr(VI), the average fold difference relative to the 0 mM Cr(VI) basal state is given. Bold values indicate the concentration at which maximum expression was observed. *, significantly different than the maximum expression level.

^b ^Mean transcript copy number/ng total RNA. SE is given in parentheses (n = 6).

^c ^Mean Fold Difference calculated by dividing the average transcript copy number in each Cr(VI) condition by the average transcript copy number in 0 mM Cr(VI). SE is given in parentheses (n = 6).

**Table 2:** Specificity of Induction of Chromate Resistance Genes^a^.

Gene	Cr(VI) 5 mM	Lead **5 μM**^b^	Arsenate **5 mM**^c^	H_2_O_2_ **5 mM**^c^
***chrL***	**63.4** **(29.7)**	0.3 (0.02)	0.6 (0.05)	12.5 (3.50)
***chrA***_6_	**50.7** **(14.5)**	0.2 (0.02)	0.8 (0.15)	3.2 (0.87)
***chrB-Cterm2***	**6.3** **(1.9)**	0.1 (0.01)	0.3 (0.03)	0.1 (0.01)
***SCHR***	**6.8** **(1.9)**	0.1 (0.01)	0.3 (0.03)	0.9 (0.12)
***chrK***	**7.2** **(1.6)**	0.1 (0.01)	0.2 (0.04)	1.0 (0.21)
***chrB-Nterm***	**16.9** **(7.1)**	0.1 (0.01)	0.4 (0.08)	0.5 (0.12)
***chrB-Cterm***	**25.4** **(4.4)**	2.6 (0.12)	5.3 (0.97)	4.9 (0.70)
***chrJ***	**92.4** **(47.2)**	0.7 (0.05)	1.7 (0.10)	6.6 (0.58)

^a ^Values shown for lead, arsenate and H_2_O_2 _represent the transcript copy number ng^-1 ^total RNA in each experimental condition relative to transcript levels in 0.2X NB and the SE (parentheses, n = 6 qRT-PCR reactions per treatment). The relative expression of each gene in 5 mM Cr(VI) is shown for comparison.

^b ^0.5 and 50 μM lead also tested with similar results

^c ^0.5 and 50 mM Arsenate and H_2_O_2 _also tested with similar results

### Potential regulatory element within the CRD

**Figure 4:**
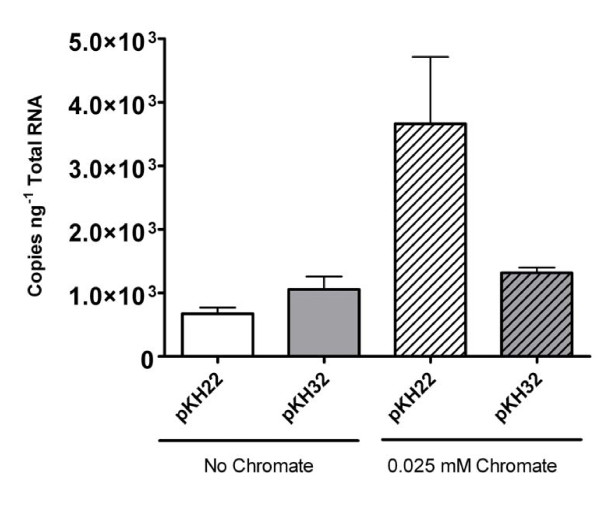
**Induction of *chrA *in D11 transformed with pKH22, pKH32**. Error bars show the standard error (n = 6 qRT-PCR reactions per treatment)

ChrB has been proposed to function as an activator of the chromate resistance determinants in *C. metallidurans *[21]. A bioinformatics analysis using protein function prediction software [37] suggested possible DNA-binding and kinase activities for ChrB-Cterm and ChrB-Nterm, respectively. In addition, proteins containing WD40 repeats, such as Arth_4252, have been associated with signal transduction and regulatory mechanisms [29,38]. To determine if *chrK*, *chrB-Nterm *and *chrB-Cterm *influence expression of *chrA*, strain D11 bearing plasmids pKH22 and pKH32 was grown in the presence and absence of chromate, and qRT-PCR was used to quantify *chrA *expression under these conditions. Expression of *chrA *was induced to higher levels by chromate in strain D11 bearing pKH22 than when the putative regulatory genes were absent (pKH32) (Figure 4). This difference is not likely to be attributable to differences in plasmid copy number provided that *chrA *expression in both strains without chromate was similar.

## Discussion

We have described a cluster of eight genes that confers chromate resistance in *Arthrobacter *sp. strain FB24 and appears to specifically respond to chromate. In other organisms, proteomic and genomic analyses revealed that chromate induces a variety of generalized stress-responsive systems, including those involved in the SOS response, DNA repair and protection against oxidative stress [39,40]. However, evidence suggests that induction of the FB24 CRD genes does not represent a general stress response. The concentrations of chromate (5 μM) that induced elevated levels of transcripts from CRD genes are 1000-fold lower than those that affect the growth rate of FB24, and the CRD genes were specifically induced in a dose-dependent manner in response to chromate exposure. Other classes of stressors (lead, arsenate or hydrogen peroxide) resulted in little or no induction of CRD genes. Furthermore, whereas other metal efflux systems, such as those in the cation diffusion facilitator (CDF) family, exhibit broad metal specificity [41,42], the lack of induction of the CRD genes by lead and arsenate supports the contention that this is a chromate-specific system.

Expression of the CRD in response to chromate was also verified at the proteomic level using tandem liquid chromatography-mass spectrometry [43]. In a global proteomic study, ORF-specific peptides were confirmed for all genes, with the exception of Arth_4249 and Arth_4250. Note that protein products were detected for the truncated genes of ChrA and ChrB (Arth_4253, 4254 and 4251). This is the first report that a SCHR gene product is synthesized in response to chromate. Although its exact function requires further experimentation, chromate-specific increases in transcript and protein abundance levels of Arth_4251 indicate that this gene, and perhaps its orthologs, plays a significant role in chromate resistance, as was seen recently with the *ywrA *and *ywrB *SCHR genes in *B. subtilis *[27]. It is important to note that SCHR in FB24 has greater sequence similarity to LCHR sequences than other SCHR sequences possibly explaining its maintenance of a chromate response. Arth_4251 may be an integral link to elucidate the evolution of chromate resistance mechanisms. It may represent a remnant precursor to the evolution of LCHR from gene duplication or the next step in evolution essential for the high chromate-resistance phenotype.

Our investigation of *Arthrobacter *sp. strain FB24 further suggests roles for three new genes (*chrJ, chrK *and *chrL*) in addition to catalytic and regulatory proteins found in those Proteobacteria and may help to explain the variability in chromate resistance levels across bacterial species. Whereas genetic studies in Proteobacteria [14,17,20,21] have pointed to the primacy of the *chrA *gene in conferring Cr(VI) resistance, the introduction of *chrA *alone into Cr(VI) sensitive strain D11 produced resistance levels that were only one-tenth of those found when the entire CRD was introduced. As of late, the *chrA *gene has only been intensively studied in two Proteobacteria, *P. aeruginosa *and *C. metallidurans*, and thus far, these systems have been the paradigm for understanding bacterial chromium resistance [13,23,44]. Recent studies with *chrA *orthologs from two additional Proteobacteria, *Shewanella *sp. strain ANA-3 [16] and *Ochrobactrum tritici *5bvl1 [17], have also demonstrated that *chrA *and neighboring genes (Figure 2) confer resistance in Cr(VI)-sensitive strains. Aguilar-Barajas et al [16] were able to recover Cr(VI)-resistance in Cr(VI)-sensitive *E. coli *and *P. aeruginosa *strains by expressing the *chr *operon from *Shewanella *sp. strain ANA-3 on a low-copy plasmid. Similar to what was shown for *Arthrobacter *FB24, though, expression of *chrA *alone resulted in lower resistance levels in *E. coli *than strains bearing the entire ANA-3 *chrBAC *operon. The ANA-3 *chrA *gene conferred chromate resistance in *P. aeruginosa*, and this phenotype was enhanced by the presence of the host *chrR *regulatory gene [16], thus emphasizing the importance of accessory genes in achieving higher levels of chromate resistance.

In the case of *Ochrobactrum*, Cr(VI)-sensitive strains transformed with a plasmid carrying the *chrA *and *chrB *genes from *TnOtChr *showed similar growth in chromate as the wild-type *O. tritici *strain. However, no additional growth advantage was provided by the presence of *chrC *and *chrF *[17]. In *C. metallidurans*, deletion of *chrC *resulted in a slight decrease in chromate resistance compared to the wild-type strain (0.3 mM chromate minimal inhibitory concentration versus 0.35 mM, respectively). In the same study, deletion of *chrF*_2 _did not affect chromate resistance levels [21]. In these organisms, it appears that *chrB *makes a significant contribution to chromate resistance, but the exact contributions made by *chrC *and *chrF *are not so apparent and may vary depending on the host strain. This is in stark contrast to the *chrJ, chrK *and *chrL *accessory genes in strain FB24, whose deletion results in a noticeable decrease in chromate resistance. A conclusion that can be drawn from these observations is that, although chromate efflux appears to be the overarching mode for resistance, the intricacies of the exact biochemical and regulatory mechanisms controlling efflux differ among bacterial strains, and these differences await full characterization.

Since most work regarding chromate efflux has been done in Proteobacteria, we were interested in whether CRD orthologs were present in strains more closely related to *Arthrobacter *sp. strain FB24. In searching for organisms with gene neighborhoods similar to the *Arthrobacter *FB24 CRD, it was discovered that other actinomycetes share a similar genetic makeup (Figure 2). *Rhodococcus *sp. RHA1 and *Nocardiodes *sp. JS614 both contain *chrK, chrB-Nterm *and *chrB-Cterm *orthologs in the near vicinity of *chrA*, while the chromate-resistant *Arthrobacter *sp. CHR15 harbors *chrJ, chrK and chrL *orthologs near *chrA *and *chrB*. The chromate resistance status of *Nocardiodes *sp. JS614 and *Rhodococcus *sp. RHA1 is not known; however, both species are known PCB degraders and are considered important environmental Actinobacteria [45-47]. The distinct genomic context between Proteobacteria and Actinobacteria suggests that functional and regulatory differences in efflux-mediated chromate resistance likely exist in distantly related taxa. This demands genetic and biochemical studies in a greater diversity of organisms in order to fully understand the breadth of physiological strategies that have evolved to confer chromium resistance.

## Conclusion

This study increases our knowledge of the genetics of chromate resistance by identifying three novel genes that play a significant role in chromate resistance in *Arthrobacter *sp. strain FB24: *chrJ*, *chrK*, and *chrL*. Future work should focus on elucidating the exact physiological function of these genes. However, our research is an important first step in characterizing potential regulatory networks controlling efflux-mediated chromate resistance. We further illustrate the value of examining the genomic context of already characterized metal resistance genes in identifying new players in metal resistance mechanisms.

## Methods

### Bacterial strains and growth conditions

Bacterial strains and plasmids used in this study are listed in Table 3. *Arthrobacter *strains were cultured in 0.1X or 0.2X nutrient broth (NB) [Difco, Sparks, MD], Luria-Bertani (LB) medium pH 7.0, or modified Xenobiotic Basal Medium (mXBM). Modified XBM contained 10 mM glycerol phosphate, 10 mM KNO_3_, 6.0 mM NH_4_NO_3_, 0.01 mM CaCl_2_, 2 ml L^-1 ^of EDTA Fe Citrate Solution [7.4 mM FeCl_3_, 11.4 mM Na_2_EDTA, 12.8 mM sodium citrate (C_6_H_5_O_7_Na_3_), 100 mM MgSO_4_, 5% NH_4_Cl_2_, 0.05 M CaCl_2_, 1.0 M NaCl, 1 M NaHCO_3_], 10 ml L^-1 ^of vitamin solution (see Jerke [48] and Additional file 4 for components), 1 ml L^-1 ^SL-7 trace elements [49], with glucose (1.7 mM) as a carbon and energy source.

**Table 3:** Bacterial strains and plasmids used in this study.

Strain or plasmid	Description	Reference
***Arthrobacter***		
FB24	Cr^R^	[6]
D11	Cr^S ^derivative of FB24	This work
***E. coli***		
JM110	*dam*^-^*dcm*^-^	Stratagene
**Plasmids**		
pAOWA10128	7.3 kb insert in pMCL200 obtained from DOE-JGI. Contains Arth_4248-Arth_4254.	DOE-JGI
pBluescript II SK+	3.0 kb, Ap^R^, *lacZ*, used for sublconing inserts prior to ligation into pART2.	Promega
pART2	4.6 kb, Km^R^, pCG100 ori, ColE1 ori, vector for expression in *Arthrobacter*	[55]
pKH11	10.6 kb PCR product from FB24 plasmid 3 (CP000457) containing Arth_4247-4255 in pBluescript II SK+	This work^a^
pKH12	Insert from pKH11 cloned into pART2	This work
pKH21	7.3 kb insert from pAOWA10128 in pBluescript II SK+	This work
pKH22	Insert from pKH21 cloned into pART2	This work
pKH32	3.7 kb *Eco*RI-*Kpn*I fragment from pKH21 cloned into pART2. Contains Arth_4248-4249.	This work
pKH42	3.8 kb *Xho*I-*Bgl*II fragment from pKH21 cloned into pART2. Contains Arth_4251-Arth_4254.	This work
pKH52	8.3 kb insert from *Mlu*I-*Bgl*II digest of pKH11 to delete Arth_4252 and Arth_4252 cloned into pART2	This work
pKH62	pKH22 digested with *Sfi*I to delete Arth_4249-Arth_4252.	This work
pKH72	pKH12 digested with *Sca*I and *Xba*I to delete Arth_4247.	This work

^a^A schematic of each construct is presented in Figure 3.

Induction of Cr(VI) resistance genes was assessed in *Arthrobacter *sp. strain FB24 cells by culturing in 150 ml NB to early mid-log phase (OD_600_, 0.3) at 30°C with shaking at 200 rpm. Cells were harvested by centrifugation, washed once with 0.2X NB and suspended in 15 ml 0.2X NB. Chromate (K_2_CrO_4_) was added to final concentrations of 0, 0.005, 0.025, 0.05, 0.1, 5, 20 or 100 mM. To test for specificity of induction, additional cultures were incubated in the presence of 0, 0.5, 5 and 50 μM PbNO_3 _in mXBM; 0, 0.5, 5 and 50 mM Na_2_HAsO_4_·7 H_2_O in 0.2X NB; and 0, 0.5, 5, 50 mM hydrogen peroxide (H_2_O_2_) in 0.2X NB. Cells were incubated for 2.5 hours at 30°C with agitation. Induction experiments with Cr(VI)-sensitive strain D11 transformed with pKH22, pKH23 and pKH24 were carried out in the same manner with the following exceptions: kanamycin was added to a concentration of 30 μg ml^-1 ^and chromate was added to one culture at a concentration of 0.025 mM.

### Generation of chromate-sensitive FB24 derivative

The lead- and chromate-sensitive mutant, D11, was generated from the resistant wild-type strain FB24 by growing cells in LB without chromate. Cultures were transferred daily by diluting cells 1:1000 into fresh media. Transfers were maintained for approximately 90 generations at 30°C with shaking at 200 rpm and then screened for cells sensitive to 75 μM lead on mXBM agar plates. Lead-sensitive colonies were then tested for Cr(VI) sensitivity on 0.1X nutrient agar (NA) plates supplemented with 0.5, 1, 2 and 5 mM K_2_CrO_4_. Loss of plasmid DNA in strain D11 was assessed by Southern hybridization and rep-PCR. Loss of the CRD genes was confirmed by PCR using gene-specific primers.

Total genomic DNA was extracted from cultures grown overnight in NB with appropriate selection. Cells were harvested by centrifugation, suspended in TE buffer, and treated with lysozyme (1 mg ml^-1^) for one hour followed by treatment with proteinase K (10 mg ml^-1^). Cells were lysed using a FastPrep instrument (Qbiogene, Carlsbad, CA) at a setting of 4 for 30 s with 0.64 cm ceramic beads. Genomic DNA was purified by phenol: chloroform: isoamyl alcohol extraction and precipitated with isopropanol [50]. DNA was digested with restriction enzymes (*Sac*I and *Xcm*I) and separated on a 0.7% agarose gel and transferred to Hybond-N+ membrane (Amersham Pharmacia, Pisscataway, NJ) using a Trans-blot semi dry transfer cell (Bio-Rad, Hercules, CA) following the manufacturer's recommendations for voltage and transfer time. A digoxigenin-labeled probe targeting the 10.6-kb CRD on *Arthrobacter *sp. strain FB24 pFB24-104 [GenBank: NC_008539] was generated by PCR with primers C42/F and C42/R (Table 4) using the TripleMaster PCR system (Eppendorf North America, Inc., Westbury, NY) according to the manufacturer's reaction mixture and cycling specifications for long-range PCR. Hybridization and chromogenic detection was carried out under high stringency conditions as described in the *DIG Application Manual for Filter Hybridization *(Roche Applied Science, Indianapolis, IN).

**Table 4:** PCR and qRT-PCR primers used in this study.

Primer	Sequence (5'→3')	Description
C42/F	**CCCAAAGCTTGGG**TCCTGCTCATCACCAGAAACTCCA	*Hind*III site, used to construct pKH11
CF2/R	**GCTCTAGAGC**AACCGCTTTCAGGCACTGTTGTTC	*Xba*I site, used to construct pKH11
**Primers for RT-PCR--asterisk denotes primer for cDNA synthesis**		
**Primer**	**Sequence (5'→3')**	**Gene**
MQO RT/A*	AGGCCTGCCCGTAGACTTTC	Arth_4255
MQO RT/B	TCTTCACCGCCGGTATGAG	Arth_4255
ChrB RT/A*	CGAGGATGAGGGATCGTTTG	Arth_4254
ChrB RT/B	TGATCCGCAGGAACATCG	Arth_4254
SP RT/F	CCCGGGAGCACTTCGACTGGA	Arth_4253
SP RT/R*	CCTGGCGCGTTCGGTTGCAT	Arth_4253
Cog4RT/F	AAGGCCTACGTCTCCAACGAACA	Arth_4252
Cog4RT/R*	ATTCTGTCGGTGACCGTGTCAGT	Arth_4252
ChrAP RT/A	GCTTCATCCTGTGCTTCTTG	Arth_4251
ChrAP RT/B*	TGTTCATGATGCCGGTACTG	Arth_4251
BP RT/F	CCTGCGCCGCTACGAACTCACCGAT	Arth_4249
BP RT/R*	GCGCTGGTGTTCGTACAGCCCGTC'	Arth_4249
ChrAC RT/A*	AAGTACAGGGCCAGGTTC	Arth_4248
ChrAC RT/B	TGCGGTCCTGTCCTATATC'	Arth_4248
Lppy RT/F	AGTGACCACGGCCATCAATTTCCA	Arth_4247
Lppy RT/R*	TCAGGGAATGATTGTGCACGGAGA	Arth_4247

### Chromate resistance determination

Starter cultures were grown overnight at 30°C in 0.2X NB with appropriate selection. Cultures for minimal inhibitory concentration (MIC) determination were diluted 1:1000 in 3 ml of 0.1X NB for chromate cultures or mXBM plus glucose for divalent cationic metals in borosilicate glass tubes and maintained at 30°C with shaking at 200 rpm. The OD_600 _was measured daily for a period of 3 days until growth stabilized. Divalent cationic metals used in MIC experiments were added as lead nitrate (Pb(NO_3_)_2_, zinc chloride (ZnCl_2_), or cadmium sulfate (CdSO_4_) at concentrations ranging from 0 to 200 μM. Cultures were prepared in triplicate for each growth or MIC experiment. D11 transformants were screened for chromate resistance by streaking single colonies onto 0.1X nutrient agar plates containing 0, 0.5, 1, 2, or 5 mM chromate.

### Sequence analysis of putative chromate efflux gene

The genome sequence is available in the GenBank database under accession numbers NC_008537 to NC_008539 and NC_008541. The genome was sequenced by the Department of Energy Joint Genome Initiative (DOE-JGI) and can be accessed at http://genome.jgi-psf.org/finished_microbes/art_f/art_f.home.html. The annotated sequence at this site was used for locating the CRD and construction of primer sequences. Multiple sequence alignment of Arth_4248 (ChrA) with other described and putative members of the CHR family of chromate efflux proteins [24] was performed using the ClustalX program and default setting for Gonnet series for protein weight matrix [51] and bootstrap Neighbor Joining tree options with 1000 resamplings. Output trees were visualized in Fig Tree http://tree.bio.ed.ac.uk/software/figtree/. Sequences were retrieved from the UniProt database [52] by conducting a search for ChrA sequences according to Diaz-Perez et al [22]. Some additional eukaryotic sequences were found by conducting a similar search of the GenBank database [53]. All short ChrA (SCHR) sequences (<350 amino acids) were excluded from the alignment. A total of 513 sequences (Additional files 1 and 2) were retrieved and aligned. Transmembrane helices were predicted using the TMHMM 2.0 server [54].

### Cloning of chromate resistance determinant

A series of constructs were created to test expression of chromate resistance in strain D11. All constructs, except for pKH62 and pKH72, were prepared by subcloning into pBluescript SK+ (Stragene, La Jolla, CA) prior to cloning into pART2 [55]. Recombinant plasmid DNA was transformed into strain D11 by electroporation as described elsewhere [56]. Ampicillin was used for selection at a concentration of 100 μg ml^-1 ^for pBluescript-derived transformants, and kanamycin was used at a concentration of 40 μg ml^-1 ^for pART2-derived transformants. Plasmids were submitted to the Purdue University Core Genomics Center for validation of insert sequences.

Plasmid pKH11 was generated by amplifying a 10.6 kb fragment bearing bases 72880 to 83464 of pFB24-104 using the TripleMaster PCR system (Eppendorf North America, Inc., Westbury, NY) according to the manufacturer's specifications and primers C42/F and C42/R. The PCR product was digested with *Hind*III and *Xba*I and ligated into pBluescript SK+ to give pKH11. Plasmid pKH21 contains a 7.3 kb insert bearing bases 74642 to 81771 from FB24-104; the insert was isolated by digesting pAOWA10128 (obtained from DOE-JGI) with *Xba*I and *Hind*III. The remaining constructs (Table 3) were generated by restriction digestion of either pKH11 or pKH21 using standard cloning procedures [50].

### Expression analysis by quantitative reverse transcriptase PCR (qRT-PCR)

Primer sequences for qRT-PCR are listed in Table 4. Total RNA was extracted from *Arthrobacter *cell pellets using the FastRNA PRO Blue Kit (MP Biomedical, Solon, OH) and treated with Turbo DNA-Free DNAse (Ambion, Austin, TX) to remove contaminating DNA. RNA concentrations were quantified by measuring the A_260 _on a Smart Spec 3000 spectrophotometer (Bio-Rad, Hercules, CA). cDNA was synthesized from 100 ng total RNA using ImProm II reverse transcriptase (RT) (Promega, Madison, WI) following the manufacturer's reaction conditions. PCR was performed using the following conditions: 98°C for 5 min, followed by 30 cycles of 94°C for 30 s, 56-58°C (depending on the primer pair) for 30 s, 72°C for 1 min, with a final extension step at 72°C for 10 min.

For real-time PCR, 1 μl of the reverse transcription reaction mixtures prepared as described above was used as the template. The PCR mixture contained 1 U of HotMaster *Taq *(Eppendorf North America, Inc., Westbury, NY), 1× HotMaster *Taq *PCR buffer with 25 mM MgCl_2_, 1% bovine serum albumin, 0.2 mM each of dNTPs, 0.25 mM each of a forward and reverse primer, SYBR Green (1:30,000; Molecular Probes, Eugene, OR) and 10 nM FITC (Sigma, St. Louis, MO) in a final volume of 25 μl. Reactions were carried out using a Bio-Rad MyIQ single-color real time PCR detection system, and data were analyzed using the MyIQ Optical System software version 2.0. Transcript copy numbers were calculated from a standard curve using known concentrations of pKH11. Plots of transcript copy number ng^-1 ^total RNA versus chromate concentration were prepared in GraphPad Prism 4 (GraphPad Software, San Diego, CA). The mean and standard errors were determined from 6 qRT-PCR reactions per chromate treatment (3 independent cultures × 2 reactions per culture). Significant differences among chromate treatments for each gene were determined by generating least square means in PROC GLIMMIX with the LS MEANS option in SAS version 9.1. Multiple comparisons were adjusted using Tukey's test. To normalize the variance of the model residuals, a negative binomial distribution was used for each set of gene expression data.

### Chromium content in chromate-exposed cells

*Arthrobacter *strains FB24 and D11 were grown to mid-log phase (OD_600_, ~0.2) in 50 ml 0.2X NB at which time four replicate cultures were amended with 2 mM chromate (final concentration). One culture per strain was incubated without chromate. All cultures were incubated for an additional 2 h. Aliquots of 40 ml of cells were harvested by centrifugation and washed 4 times with ddH_2_O. Cell pellets were solubilized in concentrated nitric acid (cHNO_3_) and heated at 95°C for 2 h. Samples were adjusted to a final concentration of 2% HNO_3 _with double distilled water and analyzed for total chromium content at the Purdue University Mass Spectrometry Center. The ^52^Cr inductively coupled argon plasma mass spectrometry (ICPMS) results were obtained using an ELEMENT-2 (ThermoFinnigan, Bremen, Germany) mass spectrometer in the medium resolution mode. The samples were introduced into the plasma using an Aridus desolvating system with a T1H nebulizer (Cetac Technologies, Omaha NE), which is used to enhance sensitivity and reduce oxide and hydride interferences. The argon sweep gas and nitrogen of the Aridus is adjusted for maximum peak height and stability using ^7^Li, ^115^In and ^238^Upeaks obtained from a multi-element standard (1 ng/ml, Merck & Co.). Chromium concentration was normalized per mg protein. Total soluble cell protein concentration was determined using the Lowry method [57] after collecting cells by centrifugation and extracting protein with 1N NaOH at 100°C. Student's t-test was used to determine statistically significant differences in the average chromium content between strains D11 and FB24 at the 95% confidence level.

## Authors' contributions

KH conceived and carried out the molecular genetic, gene expression and growth studies and performed the majority of manuscript writing. CN participated in study design and coordination, performed sequence analysis of the chromate efflux gene, alignment of chromate efflux amino acid sequences and generated the phylogenetic trees. DT participated in study design and coordination. AK participated in study design and coordination. All authors participated in drafting the manuscript. All authors read and approved the final manuscript.

## Supplementary Material

Additional file 1 **Supplemental Figure S1**. Radial phylogenetic tree of LCHR proteins generated from an alignment of 513 putative ChrA, chromate ion transport sequences (see Supplemental Table S1) using ClustalX. Neighbor Joining tree graphically viewed using the FigTree program http://tree.bio.ed.ac.uk/software/figtree/. Branched tips labeled with protein accession number followed by species name. Scale bar indicates 0.06 amino acid substitutions per site. Branches colors are fungi-brown, algae-green, Archaea-red, Proteobacteria (alpha-pink, beta-magenta, delta-blue, gamma-purple), Cyanobacteria-torquoise, Firmicutes-yellow, Actinobacteria-red and all other Bacteria-black. Click here for file

Additional file 2 **Supplemental Table S1**. Sequence accession numbers, taxa name and sequence length of putative ChrA sequences used in phylogenetic analysis. Click here for file

Additional file 3 **Supplemental Figure S2**. Operon structure analysis of the *Arthrobacter *sp. strain FB24 CRD. RT-PCR was used to determine co-transcription of the genes within the chromate resistance determinant. A: Location of primer pairs. Primer sequences are listed in table 4. Primer numbers correspond to the following primers: 1-MQO RT/A, 2-BC RT/A, 3-SP RT/F, 4-SP RT/R, 5-COG4RT/F, 6-COG4RT/R, 7-ChrAP RT/A, 8-ChrAP RT/B, 9-BP RT/R. B: RT-PCR results with listed primer pairs. C: RT-PCR products of reactions performed with primer pair 2 + 4 (lanes 2 and 3) and primer pair 5 + 8 (lanes 8 and 9). Lanes 1 and 7-100 bp PCR ruler, dark band is 1 kb; Lanes 4 and 10-no template controls; Lanes 5 and 11-No RT controls; Lanes 6 and 12 positive PCR control using pKH12 as template. Click here for file

Additional file 4 **Supplemental Table S2**. Recipe for vitamin solution added to mXBM. Click here for file
