# Supplementary figures and images for: High-level chromate resistance in Arthrobacter sp. strain FB24 requires previously uncharacterized accessory genes
Source: BMC Microbiol. 2009 Sep 16;9:199. doi: 10.1186/1471-2180-9-199 (PMC2751784; doi:10.1186/1471-2180-9-199)

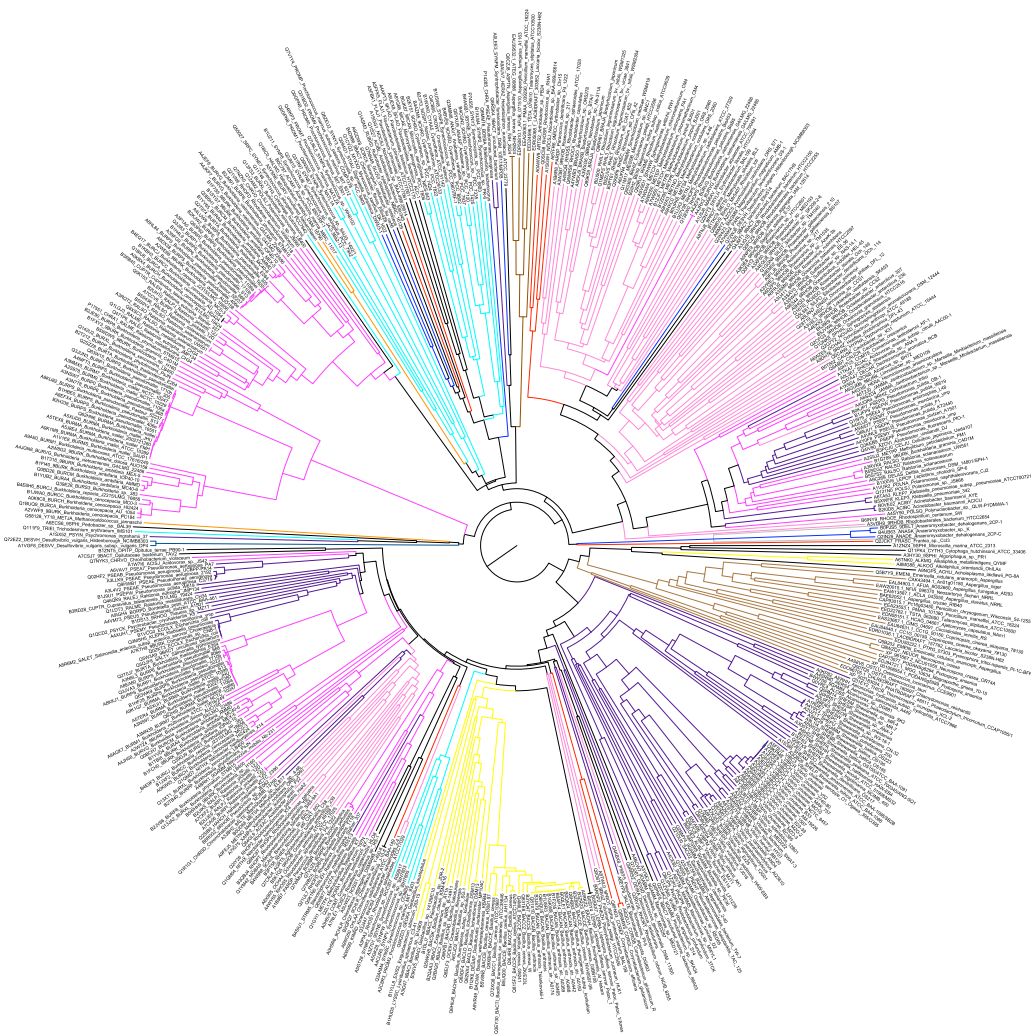

Supplement: Additional file 1 — Supplemental Figure S1. Radial phylogenetic tree of LCHR proteins generated from an alignment of 513 putative ChrA, chromate ion transport sequences (see Supplemental Table S1) using ClustalX. Neighbor Joining tree graphically viewed using the FigTree program http://tree.bio.ed.ac.uk/software/figtree/. Branched tips labeled with protein accession number followed by species name. Scale bar indicates 0.06 amino acid substitutions per site. Branches colors are fungi-brown, algae-green, Archaea-red, Proteobacteria (alpha-pink, beta-magenta, delta-blue, gamma-purple), Cyanobacteria-torquoise, Firmicutes-yellow, Actinobacteria-red and all other Bacteria-black. [file 1471-2180-9-199-S1.PDF]

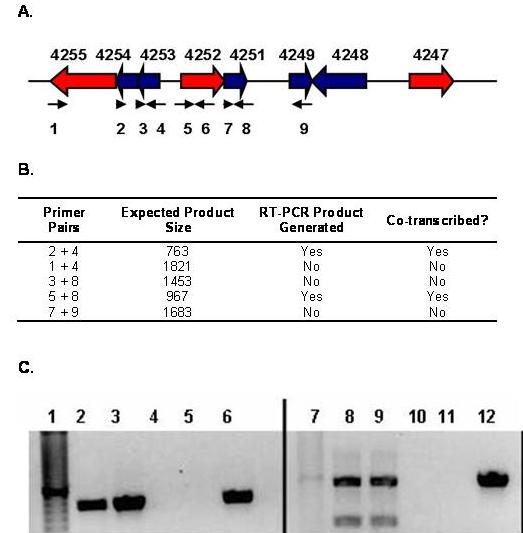

Supplement: Additional file 3 — Supplemental Figure S2. Operon structure analysis of the Arthrobacter sp. strain FB24 CRD. RT-PCR was used to determine co-transcription of the genes within the chromate resistance determinant. A: Location of primer pairs. Primer sequences are listed in table 4. Primer numbers correspond to the following primers: 1-MQO RT/A, 2-BC RT/A, 3-SP RT/F, 4-SP RT/R, 5-COG4RT/F, 6-COG4RT/R, 7-ChrAP RT/A, 8-ChrAP RT/B, 9-BP RT/R. B: RT-PCR results with listed primer pairs. C: RT-PCR products of reactions performed with primer pair 2 + 4 (lanes 2 and 3) and primer pair 5 + 8 (lanes 8 and 9). Lanes 1 and 7-100 bp PCR ruler, dark band is 1 kb; Lanes 4 and 10-no template controls; Lanes 5 and 11-No RT controls; Lanes 6 and 12 positive PCR control using pKH12 as template. [file 1471-2180-9-199-S3.JPEG]
