# Supplementary material for: High-level chromate resistance in Arthrobacter sp. strain FB24 requires previously uncharacterized accessory genes
Source: BMC Microbiol. 2009 Sep 16;9:199. doi: 10.1186/1471-2180-9-199 (PMC2751784; doi:10.1186/1471-2180-9-199)
Supplement: Additional file 2 — Supplemental Table S1. Sequence accession numbers, taxa name and sequence length of putative ChrA sequences used in phylogenetic analysis. [file 1471-2180-9-199-S2.DOC]

Supplemental Table S1: Sequence accession numbers, taxa name and sequence length of putative ChrA sequences used in phylogenetic analysis

| Accession | Phylum or Class | Genus/Species | Length (aa) |
| --- | --- | --- | --- |
| Q84GL9 | Alpha proteobacteria | *Acetobacter aceti* | 406 |
| Q7CRJ4 | Alpha proteobacteria | *Agrobacterium tumefaciens (strain C58 / ATCC 33970)* | 405 |
| A8TWL0 | Alpha proteobacteria | *Alpha proteobacterium BAL199* | 423 |
| A8TNJ8 | Alpha proteobacteria | *Alpha proteobacterium BAL199* | 448 |
| Q1YM42 | Alpha proteobacteria | *Aurantimonas sp. (strain SI85-9A1) manganese oxidizing bacterium* | 406 |
| A8IMY2 | Alpha proteobacteria | *Azorhizobium caulinodans (ATCC 43989/DSM 5975/ ORS 571)* | 462 |
| B2IIH2 | Alpha proteobacteria | *Beijerinckia indica subsp. indica (ATCC 9039 / DSM 1715 / NCIB 8712)* | 471 |
| Q89K59 | Alpha proteobacteria | *Bradyrhizobium japonicum* | 461 |
| Q9RH61 | Alpha proteobacteria | *Bradyrhizobium japonicum* | 461 |
| A5EKJ9 | Alpha proteobacteria | *Bradyrhizobium sp. (strain BTAi1 / ATCC BAA-1182)* | 463 |
| A4YWB3 | Alpha proteobacteria | *Bradyrhizobium sp. (strain ORS278)* | 463 |
| B4W8R8 | Alpha proteobacteria | *Brevundimonas sp. BAL3* | 410 |
| Q9A997 | Alpha proteobacteria | *Caulobacter crescentus (Caulobacter vibrioides)* | 426 |
| B0T802 | Alpha proteobacteria | *Caulobacter sp. (strain K31)* | 431 |
| A8LRN7 | Alpha proteobacteria | *Dinoroseobacter shibae (strain DFL 12)* | 417 |
| Q2N7T3 | Alpha proteobacteria | *Erythrobacter litoralis (strain HTCC2594)* | 423 |
| Q0G671 | Alpha proteobacteria | *Fulvimarina pelagi HTCC2506* | 396 |
| Q0G369 | Alpha proteobacteria | *Fulvimarina pelagi HTCC2506* | 434 |
| A9DH76 | Alpha proteobacteria | *Hoeflea phototrophica DFL-43* | 444 |
| Q0C4N9 | Alpha proteobacteria | *Hyphomonas neptunium (strain ATCC 15444)* | 475 |
| Q28T19 | Alpha proteobacteria | *Jannaschia sp. (strain CCS1)* | 418 |
| A0NPE9 | Alpha proteobacteria | *Labrenzia aggregata IAM 12614* | 442 |
| A3V8L3 | Alpha proteobacteria | *Loktanella vestfoldensis SKA53* | 416 |
| Q2W4Z3 | Alpha proteobacteria | *Magnetospirillum magneticum (strain AMB-1/ATCC 700264)* | 452 |
| Q11M17 | Alpha proteobacteria | *Mesorhizobium sp. (strain BNC1)* | 466 |
| A7WDW4 | Alpha proteobacteria | *Methylobacterium chloromethanicum CM4* | 467 |
| A7W950 | Alpha proteobacteria | *Methylobacterium chloromethanicum CM4* | 471 |
| A9W2S0 | Alpha proteobacteria | *Methylobacterium extorquens (strain PA1)* | 467 |
| A9FEJ5 | Alpha proteobacteria | *Methylobacterium nodulans ORS 2060* | 411 |
| A9H2A3 | Alpha proteobacteria | *Methylobacterium nodulans ORS 2060* | 469 |
| A9HXC0 | Alpha proteobacteria | *Methylobacterium nodulans ORS 2060* | 469 |
| B1ZD95 | Alpha proteobacteria | *Methylobacterium populi (strain ATCC BAA-705 / NCIMB 13946 / BJ001)* | 469 |
| B1ZJF1 | Alpha proteobacteria | *Methylobacterium populi (strain ATCC BAA-705 / NCIMB 13946 / BJ001)* | 469 |
| B1LUX3 | Alpha proteobacteria | *Methylobacterium radiotolerans (strain ATCC 27329 / DSM 1819 / JCM 2831)* | 460 |
| B0UD68 | Alpha proteobacteria | *Methylobacterium sp. (strain 4-46)* | 398 |
| B0UPK6 | Alpha proteobacteria | *Methylobacterium sp. (strain 4-46)* | 466 |
| B1TKC9 | Alpha proteobacteria | *Methylocella silvestris BL2* | 467 |
| Q1QMD7 | Alpha proteobacteria | *Nitrobacter hamburgensis (strain X14 / DSM 10229)* | 396 |
| Q1QM04 | Alpha proteobacteria | *Nitrobacter hamburgensis (strain X14 / DSM 10229)* | 430 |
| A3WXG6 | Alpha proteobacteria | *Nitrobacter sp. Nb-311A* | 463 |
| A4BRK1 | Alpha proteobacteria | *Nitrococcus mobilis Nb-231* | 409 |
| Q2G7Y2 | Alpha proteobacteria | *Novosphingobium aromaticivorans (strain DSM 12444)* | 443 |
| A9E544 | Alpha proteobacteria | *Oceanibulbus indolifex HEL-45* | 414 |
| A3U411 | Alpha proteobacteria | *Oceanicola batsensis HTCC2597* | 413 |
| Q2CFG3 | Alpha proteobacteria | *Oceanicola granulosus HTCC2516* | 414 |
| A6X805 | Alpha proteobacteria | *Ochrobactrum anthropi (strain ATCC 49188 / DSM 6882 / NCTC 12168)* | 407 |
| A6WZE5 | Alpha proteobacteria | *Ochrobactrum anthropi (strain ATCC 49188 / DSM 6882 / NCTC 12168)* | 432 |
| A4UQR4 | Alpha proteobacteria | *Ochrobactrum tritici* | 458 |
| B5K1Q5 | Alpha proteobacteria | *Octadecabacter antarcticus 238* | 400 |
| B5JYW8 | Alpha proteobacteria | *Octadecabacter antarcticus 238* | 407 |
| B5J8K9 | Alpha proteobacteria | *Octadecabacter antarcticus 307* | 407 |
| A1B9B7 | Alpha proteobacteria | *Paracoccus denitrificans (strain Pd 1222)* | 405 |
| A1B6R8 | Alpha proteobacteria | *Paracoccus denitrificans (strain Pd 1222)* | 454 |
| A7HWK4 | Alpha proteobacteria | *Parvibaculum lavamentivorans (strain DS-1 / DSM 13023 / NCIMB 13966)* | 443 |
| A9EM02 | Alpha proteobacteria | *Phaeobacter gallaeciensis 2.10* | 445 |
| A9FLJ8 | Alpha proteobacteria | *Phaeobacter gallaeciensis BS107* | 432 |
| B4RGP2 | Alpha proteobacteria | *Phenylobacterium zucineum (strain HLK1)* | 400 |
| B6R0G2 | Alpha proteobacteria | *Pseudovibrio sp. JE062* | 408 |
| Q2K6P9 | Alpha proteobacteria | *Rhizobium etli (strain CFN 42 / ATCC 51251)* | 473 |
| B3PT58 | Alpha proteobacteria | *Rhizobium etli (strain CIAT 652)* | 410 |
| B3PT61 | Alpha proteobacteria | *Rhizobium etli (strain CIAT 652)* | 473 |
| B5ZUV5 | Alpha proteobacteria | *Rhizobium leguminosarum bv. trifolii (strain WSM2304)* | 473 |
| B0J500 | Alpha proteobacteria | *Rhizobium leguminosarum bv. trifolii WSM1325* | 473 |
| Q1MEF5 | Alpha proteobacteria | *Rhizobium leguminosarum bv. viciae (strain 3841)* | 473 |
| Q98BV9 | Alpha proteobacteria | *Rhizobium loti (Mesorhizobium loti)* | 463 |
| Q92RU1 | Alpha proteobacteria | *Rhizobium meliloti (Sinorhizobium meliloti)* | 480 |
| Q6WRS5 | Alpha proteobacteria | *Rhodobacter capsulatus (Rhodopseudomonas capsulata)* | 449 |
| Q3J447 | Alpha proteobacteria | *Rhodobacter sphaeroides (strain ATCC 17023 / 2.4.1 / NCIB 8253 / DSM 158)* | 392 |
| A4WY65 | Alpha proteobacteria | *Rhodobacter sphaeroides (strain ATCC 17025 / ATH 2.4.3)* | 405 |
| A4WQI4 | Alpha proteobacteria | *Rhodobacter sphaeroides (strain ATCC 17025 / ATH 2.4.3)* | 461 |
| A3PIA2 | Alpha proteobacteria | *Rhodobacter sphaeroides (strain ATCC 17029 / ATH 2.4.9)* | 392 |
| A3JQ18 | Alpha proteobacteria | *Rhodobacterales bacterium HTCC2150* | 428 |
| Q0FCZ4 | Alpha proteobacteria | *Rhodobacterales bacterium HTCC2255* | 423 |
| A3VDH9 | Alpha proteobacteria | *Rhodobacterales bacterium HTCC2654* | 405 |
| Q6NC87 | Alpha proteobacteria | *Rhodopseudomonas palustris* | 384 |
| Q07V38 | Alpha proteobacteria | *Rhodopseudomonas palustris (strain BisA53)* | 472 |
| Q13EW0 | Alpha proteobacteria | *Rhodopseudomonas palustris (strain BisB5)* | 462 |
| Q2IY26 | Alpha proteobacteria | *Rhodopseudomonas palustris (strain HaA2)* | 415 |
| B3QBJ4 | Alpha proteobacteria | *Rhodopseudomonas palustris (strain TIE-1)* | 407 |
| B6INY9 | Alpha proteobacteria | *Rhodospirillum centenum (strain ATCC 51521 / SW)* | 451 |
| Q2RV35 | Alpha proteobacteria | *Rhodospirillum rubrum (strain ATCC 11170 / NCIB 8255)* | 416 |
| Q167I0 | Alpha proteobacteria | *Roseobacter denitrificans (strain ATCC 33942 / OCh 114) (Erythrobacter sp. (strain OCh 114)) (Roseobacter denitrificans)* | 415 |
| A9HQI3 | Alpha proteobacteria | *Roseobacter litoralis Och 149* | 420 |
| A6FRJ9 | Alpha proteobacteria | *Roseobacter sp. AzwK-3b* | 419 |
| A4EKX2 | Alpha proteobacteria | *Roseobacter sp. CCS2* | 417 |
| A3XF60 | Alpha proteobacteria | *Roseobacter sp. MED193* | 420 |
| A4EQU8 | Alpha proteobacteria | *Roseobacter sp. SK209-2-6* | 422 |
| A3SMI0 | Alpha proteobacteria | *Roseovarius nubinhibens ISM* | 413 |
| A3VWE3 | Alpha proteobacteria | *Roseovarius sp. 217* | 417 |
| A3W3B7 | Alpha proteobacteria | *Roseovarius sp. 217* | 454 |
| Q0FH12 | Alpha proteobacteria | *Roseovarius sp. HTCC2601* | 416 |
| A6DZP5 | Alpha proteobacteria | *Roseovarius sp. TM1035* | 417 |
| A3K9E8 | Alpha proteobacteria | *Sagittula stellata E-37* | 420 |
| Q5LS61 | Alpha proteobacteria | *Silicibacter pomeroyi* | 456 |
| Q1GGN4 | Alpha proteobacteria | *Silicibacter sp. (strain TM1040)* | 428 |
| A6UFN4 | Alpha proteobacteria | *Sinorhizobium medicae (strain WSM419) (Ensifer medicae)* | 469 |
| A5V9D1 | Alpha proteobacteria | *Sphingomonas wittichii (strain RW1 / DSM 6014 / JCM 10273)* | 465 |
| Q1GNK8 | Alpha proteobacteria | *Sphingopyxis alaskensis (Sphingomonas alaskensis)* | 449 |
| A3SAY0 | Alpha proteobacteria | *Sulfitobacter sp. EE-36* | 418 |
| A3SWA7 | Alpha proteobacteria | *Sulfitobacter sp. NAS-14.1* | 418 |
| A7IG75 | Alpha proteobacteria | *Xanthobacter autotrophicus (strain ATCC BAA-1158 / Py2)* | 426 |
| A1TKA7 | Beta proteobacteria | *Acidovorax avenae subsp. citrulli (strain AAC00-1)* | 451 |
| A1W7I6 | Beta proteobacteria | *Acidovorax sp. (strain JS42)* | 407 |
| Q6WB77 | Beta proteobacteria | *Alcaligenes faecalis* | 408 |
| A1K9S4 | Beta proteobacteria | *Azoarcus sp. (strain BH72)* | 455 |
| Q2L1T6 | Beta proteobacteria | *Bordetella avium (strain 197N)* | 410 |
| Q7WMU4 | Beta proteobacteria | *Bordetella bronchiseptica (Alcaligenes bronchisepticus)* | 441 |
| Q7WBC5 | Beta proteobacteria | *Bordetella parapertussis* | 409 |
| Q7VUZ5 | Beta proteobacteria | *Bordetella pertussis* | 409 |
| A9IGH4 | Beta proteobacteria | *Bordetella petrii (strain ATCC BAA-461 / DSM 12804 / CCUG 43448)* | 404 |
| Q0B5H1 | Beta proteobacteria | *Burkholderia ambifaria (strain ATCC BAA-244 / AMMD) (Burkholderia cepacia (strain AMMD))* | 392 |
| Q0BD26 | Beta proteobacteria | *Burkholderia ambifaria (strain ATCC BAA-244 / AMMD) (Burkholderia cepacia (strain AMMD))* | 402 |
| Q0BCH7 | Beta proteobacteria | *Burkholderia ambifaria (strain ATCC BAA-244 / AMMD) (Burkholderia cepacia (strain AMMD))* | 425 |
| B1YVD4 | Beta proteobacteria | *Burkholderia ambifaria (strain MC40-6)* | 388 |
| B1YY65 | Beta proteobacteria | *Burkholderia ambifaria (strain MC40-6)* | 392 |
| B1YUB2 | Beta proteobacteria | *Burkholderia ambifaria (strain MC40-6)* | 423 |
| B1F7S5 | Beta proteobacteria | *Burkholderia ambifaria IOP40-10* | 392 |
| B1FCH0 | Beta proteobacteria | *Burkholderia ambifaria IOP40-10* | 419 |
| B1FI40 | Beta proteobacteria | *Burkholderia ambifaria IOP40-10* | 423 |
| B1SYG7 | Beta proteobacteria | *Burkholderia ambifaria MEX-5* | 392 |
| B1TBI8 | Beta proteobacteria | *Burkholderia ambifaria MEX-5* | 419 |
| B1T318 | Beta proteobacteria | *Burkholderia ambifaria MEX-5* | 423 |
| Q1BIU6 | Beta proteobacteria | *Burkholderia cenocepacia (strain AU 1054)* | 390 |
| Q1BUQ9 | Beta proteobacteria | *Burkholderia cenocepacia (strain AU 1054)* | 433 |
| A0KBX4 | Beta proteobacteria | *Burkholderia cenocepacia (strain HI2424)* | 390 |
| A0K9C8 | Beta proteobacteria | *Burkholderia cenocepacia (strain HI2424)* | 401 |
| A0K9W5 | Beta proteobacteria | *Burkholderia cenocepacia (strain HI2424)* | 403 |
| B1KBH3 | Beta proteobacteria | *Burkholderia cenocepacia (strain MC0-3)* | 390 |
| B1JXF2 | Beta proteobacteria | *Burkholderia cenocepacia (strain MC0-3)* | 403 |
| B1JWA0 | Beta proteobacteria | *Burkholderia cenocepacia (strain MC0-3)* | 422 |
| A2VWF9 | Beta proteobacteria | *Burkholderia cenocepacia PC184* | 433 |
| B4EQ17 | Beta proteobacteria | *Burkholderia cepacia (strain J2315 / LMG 16656) (Burkholderia cenocepacia (strain J2315))* | 390 |
| B4E6H6 | Beta proteobacteria | *Burkholderia cepacia (strain J2315 / LMG 16656) (Burkholderia cenocepacia (strain J2315))* | 402 |
| B4E9F3 | Beta proteobacteria | *Burkholderia cepacia (strain J2315 / LMG 16656) (Burkholderia cenocepacia (strain J2315))* | 403 |
| A2W8G2 | Beta proteobacteria | *Burkholderia dolosa AUO158* | 400 |
| A2WE68 | Beta proteobacteria | *Burkholderia dolosa AUO158* | 400 |
| A2W7Z4 | Beta proteobacteria | *Burkholderia dolosa AUO158* | 420 |
| B1FXY3 | Beta proteobacteria | *Burkholderia graminis C4D1M* | 402 |
| B1G789 | Beta proteobacteria | *Burkholderia graminis C4D1M* | 442 |
| Q62HI9 | Beta proteobacteria | *Burkholderia mallei (Pseudomonas mallei)* | 401 |
| Q62HN6 | Beta proteobacteria | *Burkholderia mallei (Pseudomonas mallei)* | 402 |
| A2S978 | Beta proteobacteria | *Burkholderia mallei (strain NCTC 10229)* | 397 |
| A3MMX0 | Beta proteobacteria | *Burkholderia mallei (strain NCTC 10247)* | 397 |
| A3MN39 | Beta proteobacteria | *Burkholderia mallei (strain NCTC 10247)* | 401 |
| A1V1E8 | Beta proteobacteria | *Burkholderia mallei (strain SAVP1)* | 422 |
| A5TER4 | Beta proteobacteria | *Burkholderia mallei 2002721280* | 401 |
| A5TEX8 | Beta proteobacteria | *Burkholderia mallei 2002721280* | 422 |
| A9K1G7 | Beta proteobacteria | *Burkholderia mallei ATCC 10399* | 401 |
| A9K1M9 | Beta proteobacteria | *Burkholderia mallei ATCC 10399* | 422 |
| A5J8E4 | Beta proteobacteria | *Burkholderia mallei FMH* | 422 |
| A5XUI1 | Beta proteobacteria | *Burkholderia mallei JHU* | 401 |
| A5XUD0 | Beta proteobacteria | *Burkholderia mallei JHU* | 422 |
| A9AII0 | Beta proteobacteria | *Burkholderia multivorans (strain ATCC 17616 / 249)* | 400 |
| A9AGK7 | Beta proteobacteria | *Burkholderia multivorans (strain ATCC 17616 / 249)* | 403 |
| B2JM91 | Beta proteobacteria | *Burkholderia phymatum (strain DSM 17167 / STM815)* | 394 |
| B2JE90 | Beta proteobacteria | *Burkholderia phymatum (strain DSM 17167 / STM815)* | 411 |
| B2JV66 | Beta proteobacteria | *Burkholderia phymatum (strain DSM 17167 / STM815)* | 412 |
| B2T212 | Beta proteobacteria | *Burkholderia phytofirmans (strain DSM 17436 / PsJN)* | 402 |
| B2TB40 | Beta proteobacteria | *Burkholderia phytofirmans (strain DSM 17436 / PsJN)* | 405 |
| Q63P92 | Beta proteobacteria | *Burkholderia pseudomallei* | 396 |
| Q63W15 | Beta proteobacteria | *Burkholderia pseudomallei* | 397 |
| Q63WK9 | Beta proteobacteria | *Burkholderia pseudomallei* | 401 |
| A3P1A0 | Beta proteobacteria | *Burkholderia pseudomallei (strain 1106a)* | 396 |
| A3NSW7 | Beta proteobacteria | *Burkholderia pseudomallei (strain 1106a)* | 397 |
| A3NS97 | Beta proteobacteria | *Burkholderia pseudomallei (strain 1106a)* | 401 |
| Q3JI83 | Beta proteobacteria | *Burkholderia pseudomallei (strain 1710b)* | 396 |
| Q3JUN3 | Beta proteobacteria | *Burkholderia pseudomallei (strain 1710b)* | 397 |
| Q3JVA3 | Beta proteobacteria | *Burkholderia pseudomallei (strain 1710b)* | 401 |
| A3NFG9 | Beta proteobacteria | *Burkholderia pseudomallei (strain 668)* | 396 |
| A3N778 | Beta proteobacteria | *Burkholderia pseudomallei (strain 668)* | 397 |
| A3N6L3 | Beta proteobacteria | *Burkholderia pseudomallei (strain 668)* | 401 |
| B2KJW2 | Beta proteobacteria | *Burkholderia pseudomallei 1655* | 376 |
| B2H338 | Beta proteobacteria | *Burkholderia pseudomallei 1655* | 402 |
| A4LUW9 | Beta proteobacteria | *Burkholderia pseudomallei 305* | 376 |
| A4MFT3 | Beta proteobacteria | *Burkholderia pseudomallei 305* | 397 |
| A8DX38 | Beta proteobacteria | *Burkholderia pseudomallei 406e* | 396 |
| A8EA60 | Beta proteobacteria | *Burkholderia pseudomallei 406e* | 401 |
| A8EFX4 | Beta proteobacteria | *Burkholderia pseudomallei 406e* | 422 |
| A8HL84 | Beta proteobacteria | *Burkholderia pseudomallei Pasteur 52237* | 396 |
| A8KKJ1 | Beta proteobacteria | *Burkholderia pseudomallei Pasteur 52237* | 401 |
| A8KU82 | Beta proteobacteria | *Burkholderia pseudomallei Pasteur 52237* | 402 |
| B1H4L9 | Beta proteobacteria | *Burkholderia pseudomallei S13* | 396 |
| B1HF29 | Beta proteobacteria | *Burkholderia pseudomallei S13* | 401 |
| B1HBE0 | Beta proteobacteria | *Burkholderia pseudomallei S13* | 422 |
| Q39DJ9 | Beta proteobacteria | *Burkholderia sp. (strain 383) (Burkholderia cepacia (strain ATCC 17760 / NCIB 9086 / R18194))* | 403 |
| Q39E28 | Beta proteobacteria | *Burkholderia sp. (strain 383) (Burkholderia cepacia (strain ATCC 17760 / NCIB 9086 / R18194))* | 423 |
| B5WDW5 | Beta proteobacteria | *Burkholderia sp. H160* | 402 |
| B5WHH9 | Beta proteobacteria | *Burkholderia sp. H160* | 405 |
| Q2T974 | Beta proteobacteria | *Burkholderia thailandensis (strain E264 / ATCC 700388 / DSM 13276 / CIP 106301)* | 396 |
| Q2SZZ8 | Beta proteobacteria | *Burkholderia thailandensis (strain E264 / ATCC 700388 / DSM 13276 / CIP 106301)* | 402 |
| Q2T0J7 | Beta proteobacteria | *Burkholderia thailandensis (strain E264 / ATCC 700388 / DSM 13276 / CIP 106301)* | 403 |
| A4JKP7 | Beta proteobacteria | *Burkholderia vietnamiensis (strain G4 / LMG 22486) (Burkholderia cepacia (strain R1808))* | 392 |
| A4JEF6 | Beta proteobacteria | *Burkholderia vietnamiensis (strain G4 / LMG 22486) (Burkholderia cepacia (strain R1808))* | 395 |
| A4JGN8 | Beta proteobacteria | *Burkholderia vietnamiensis (strain G4 / LMG 22486) (Burkholderia cepacia (strain R1808))* | 402 |
| A4JH58 | Beta proteobacteria | *Burkholderia vietnamiensis (strain G4 / LMG 22486) (Burkholderia cepacia (strain R1808))* | 405 |
| A4JEF9 | Beta proteobacteria | *Burkholderia vietnamiensis (strain G4 / LMG 22486) (Burkholderia cepacia (strain R1808))* | 472 |
| A4JRX3 | Beta proteobacteria | *Burkholderia vietnamiensis (strain G4 / LMG 22486) (Burkholderia cepacia (strain R1808))* | 474 |
| Q13FS3 | Beta proteobacteria | *Burkholderia xenovorans (strain LB400)* | 398 |
| Q142U2 | Beta proteobacteria | *Burkholderia xenovorans (strain LB400)* | 402 |
| Q13JA2 | Beta proteobacteria | *Burkholderia xenovorans (strain LB400)* | 412 |
| Q13XT1 | Beta proteobacteria | *Burkholderia xenovorans (strain LB400)* | 430 |
| Q7NZK0 | Beta proteobacteria | *Chromobacterium violaceum* | 393 |
| Q7NYK3 | Beta proteobacteria | *Chromobacterium violaceum* | 437 |
| A7K7H9 | Beta proteobacteria | *Comamonas sp. CNB-1* | 401 |
| A0H7V5 | Beta proteobacteria | *Comamonas testosteroni KF-1* | 452 |
| A0H6V5 | Beta proteobacteria | *Comamonas testosteroni KF-1* | 482 |
| B3RB45 | Beta proteobacteria | *Cupriavidus taiwanensis (strain R1 / LMG 19424) (Ralstonia taiwanensis (strain LMG 19424))* | 400 |
| B3RD28 | Beta proteobacteria | *Cupriavidus taiwanensis (strain R1 / LMG 19424) (Ralstonia taiwanensis (strain LMG 19424))* | 407 |
| Q47A47 | Beta proteobacteria | *Dechloromonas aromatica (strain RCB)* | 454 |
| A9C269 | Beta proteobacteria | *Delftia acidovorans (strain DSM 14801 / SPH-1)* | 452 |
| A4G3I9 | Beta proteobacteria | *Herminiimonas arsenicoxydans* | 456 |
| A6T2J0 | Beta proteobacteria | *Janthinobacterium sp. (strain Marseille) (Minibacterium massiliensis)* | 457 |
| A6SZD4 | Beta proteobacteria | *Janthinobacterium sp. (strain Marseille) (Minibacterium massiliensis)* | 477 |
| B1XXV6 | Beta proteobacteria | *Leptothrix cholodnii (strain ATCC 51168 / LMG 8142 / SP-6) (Leptothrix discophora (strain SP-6))* | 456 |
| A6GUM6 | Beta proteobacteria | *Limnobacter sp. MED105* | 453 |
| A2SIU2 | Beta proteobacteria | *Methylibium petroleiphilum (strain PM1)* | 445 |
| Q1GYI1 | Beta proteobacteria | *Methylobacillus flagellatus (strain KT / ATCC 51484 / DSM 6875)* | 404 |
| A1VUR2 | Beta proteobacteria | *Polaromonas naphthalenivorans (strain CJ2)* | 454 |
| Q12AM2 | Beta proteobacteria | *Polaromonas sp. (strain JS666 / ATCC BAA-500)* | 399 |
| Q121N0 | Beta proteobacteria | *Polaromonas sp. (strain JS666 / ATCC BAA-500)* | 456 |
| A4SY90 | Beta proteobacteria | *Polynucleobacter sp. (strain QLW-P1DMWA-1)* | 442 |
| Q0K153 | Beta proteobacteria | *Ralstonia eutropha (strain ATCC 17699 / H16 / DSM 428 / Stanier 337) (Cupriavidus necator (strain ATCC 17699 / H16 / DSM 428 / Stanier 337))* | 400 |
| Q0K1N0 | Beta proteobacteria | *Ralstonia eutropha (strain ATCC 17699 / H16 / DSM 428 / Stanier 337) (Cupriavidus necator (strain ATCC 17699 / H16 / DSM 428 / Stanier 337))* | 410 |
| Q471P6 | Beta proteobacteria | *Ralstonia eutropha (strain JMP134) (Alcaligenes eutrophus)* | 401 |
| Q46QK9 | Beta proteobacteria | *Ralstonia eutropha (strain JMP134) (Alcaligenes eutrophus)* | 413 |
| Q1LGJ3 | Beta proteobacteria | *Ralstonia metallidurans (strain CH34 / ATCC 43123 / DSM 2839)* | 390 |
| P17551 | Beta proteobacteria | *Ralstonia metallidurans (strain CH34 / ATCC 43123 / DSM 2839)* | 401 |
| Q1LDT3 | Beta proteobacteria | *Ralstonia metallidurans (strain CH34 / ATCC 43123 / DSM 2839)* | 408 |
| B2UH10 | Beta proteobacteria | *Ralstonia pickettii (strain 12J)* | 390 |
| B2UJE9 | Beta proteobacteria | *Ralstonia pickettii (strain 12J)* | 408 |
| B2UIK5 | Beta proteobacteria | *Ralstonia pickettii (strain 12J)* | 411 |
| A7CCV6 | Beta proteobacteria | *Ralstonia pickettii 12D* | 390 |
| A7CA06 | Beta proteobacteria | *Ralstonia pickettii 12D* | 408 |
| A7CKF2 | Beta proteobacteria | *Ralstonia pickettii 12D* | 411 |
| B5S436 | Beta proteobacteria | *Ralstonia solanacearum (Pseudomonas solanacearum)* | 390 |
| B5S9Y4 | Beta proteobacteria | *Ralstonia solanacearum (Pseudomonas solanacearum)* | 390 |
| Q8XSC3 | Beta proteobacteria | *Ralstonia solanacearum (Pseudomonas solanacearum)* | 401 |
| B5S7S2 | Beta proteobacteria | *Ralstonia solanacearum (Pseudomonas solanacearum)* | 448 |
| B5SEG2 | Beta proteobacteria | *Ralstonia solanacearum (Pseudomonas solanacearum)* | 448 |
| A3RQT2 | Beta proteobacteria | *Ralstonia solanacearum UW551* | 390 |
| A3RVK8 | Beta proteobacteria | *Ralstonia solanacearum UW551* | 448 |
| Q21VN9 | Beta proteobacteria | *Rhodoferax ferrireducens (strain DSM 15236 / ATCC BAA-621 / T118)* | 397 |
| Q21UL7 | Beta proteobacteria | *Rhodoferax ferrireducens (strain DSM 15236 / ATCC BAA-621 / T118)* | 417 |
| B1DS13 | Beta proteobacteria | *Thauera sp. MZ1T* | 443 |
| Q2IN29 | Delta proteobacteria | *Anaeromyxobacter dehalogenans (strain 2CP-C)* | 416 |
| B0PJS5 | Delta proteobacteria | *Anaeromyxobacter dehalogenans 2CP-1* | 444 |
| B4UB63 | Delta proteobacteria | *Anaeromyxobacter sp. (strain K)* | 430 |
| Q6MM16 | Delta proteobacteria | *Bdellovibrio bacteriovorus* | 378 |
| Q72WJ6 | Delta proteobacteria | *Desulfovibrio vulgaris (strain Hildenborough / ATCC 29579 / NCIMB 8303)* | 445 |
| Q72EZ2 | Delta proteobacteria | *Desulfovibrio vulgaris (strain Hildenborough / ATCC 29579 / NCIMB 8303)* | 450 |
| A1VGF8 | Delta proteobacteria | *Desulfovibrio vulgaris subsp. vulgaris (strain DP4)* | 450 |
| Q1CYQ2 | Delta proteobacteria | *Myxococcus xanthus (strain DK 1622)* | 403 |
| A9GDW8 | Delta proteobacteria | *Sorangium cellulosum (strain So ce56) (Polyangium cellulosum (strain So ce56))* | 400 |
| Q094G1 | Delta proteobacteria | *Stigmatella aurantiaca DW4/3-1* | 406 |
| A0LEE3 | Delta proteobacteria | *Syntrophobacter fumaroxidans (strain DSM 10017 / MPOB)* | 428 |
| B2I0D8 | Gamma proteobacteria | *Acinetobacter baumannii (strain ACICU)* | 449 |
| B0VE02 | Gamma proteobacteria | *Acinetobacter baumannii (strain AYE)* | 449 |
| A0KP82 | Gamma proteobacteria | *Aeromonas hydrophila subsp. hydrophila (strain ATCC 7966 / NCIB 9240)* | 383 |
| A4SRM0 | Gamma proteobacteria | *Aeromonas salmonicida (strain A449)* | 384 |
| Q0VRL4 | Gamma proteobacteria | *Alcanivorax borkumensis (strain SK2 / ATCC 700651 / DSM 11573)* | 383 |
| B4WY09 | Gamma proteobacteria | *Alcanivorax sp. DG881* | 378 |
| B6EGE2 | Gamma proteobacteria | *Aliivibrio salmonicida (strain LFI1238) (Vibrio salmonicida (strain LFI1238))* | 383 |
| A0Y5F6 | Gamma proteobacteria | *Alteromonadales bacterium TW-7* | 393 |
| Q4IV90 | Gamma proteobacteria | *Azotobacter vinelandii DJ* | 453 |
| B3PCJ0 | Gamma proteobacteria | *Cellvibrio japonicus (strain Ueda107)* | 450 |
| Q1R1G1 | Gamma proteobacteria | *Chromohalobacter salexigens (strain DSM 3043 / ATCC BAA-138 / NCIMB 13768)* | 404 |
| B1VCG0 | Gamma proteobacteria | *Escherichia coli* | 389 |
| Q0ZKT3 | Gamma proteobacteria | *Escherichia coli* | 401 |
| Q2SIB9 | Gamma proteobacteria | *Hahella chejuensis (strain KCTC 2396)* | 403 |
| Q3MSH5 | Gamma proteobacteria | *Klebsiella pneumoniae* | 401 |
| B5XWF8 | Gamma proteobacteria | *Klebsiella pneumoniae (strain 342)* | 455 |
| A6TA53 | Gamma proteobacteria | *Klebsiella pneumoniae subsp. pneumoniae (strain ATCC 700721 / MGH 78578)* | 455 |
| A6EYP8 | Gamma proteobacteria | *Marinobacter algicola DG893* | 397 |
| A1U5J6 | Gamma proteobacteria | *Marinobacter aquaeolei (strain VT8) (ATCC 700491 / DSM 11845)* | 483 |
| A3JBH2 | Gamma proteobacteria | *Marinobacter sp. ELB17* | 401 |
| A6VTB1 | Gamma proteobacteria | *Marinomonas sp. (strain MWYL1)* | 404 |
| A3Y5E3 | Gamma proteobacteria | *Marinomonas sp. MED121* | 399 |
| A6FGW8 | Gamma proteobacteria | *Moritella sp. PE36* | 383 |
| Q2BMY4 | Gamma proteobacteria | *Neptuniibacter caesariensis* | 380 |
| Q1N595 | Gamma proteobacteria | *Oceanobacter sp. RED65* | 377 |
| Q6LIP5 | Gamma proteobacteria | *Photobacterium profundum (strain SS9))* | 388 |
| Q1Z2P2 | Gamma proteobacteria | *Photobacterium profundum 3TCK* | 388 |
| Q2C4K2 | Gamma proteobacteria | *Photobacterium sp. SKA34* | 383 |
| Q3IJM3 | Gamma proteobacteria | *Pseudoalteromonas haloplanktis (strain TAC 125)* | 383 |
| A4CE80 | Gamma proteobacteria | *Pseudoalteromonas tunicata D2* | 368 |
| Q9HWB1 | Gamma proteobacteria | *Pseudomonas aeruginosa* | 401 |
| P14285 | Gamma proteobacteria | *Pseudomonas aeruginosa* | 416 |
| A6VAV7 | Gamma proteobacteria | *Pseudomonas aeruginosa (strain PA7)* | 401 |
| Q02HF2 | Gamma proteobacteria | *Pseudomonas aeruginosa (strain UCBPP-PA14)* | 401 |
| A3LLK9 | Gamma proteobacteria | *Pseudomonas aeruginosa 2192* | 401 |
| A3L4V2 | Gamma proteobacteria | *Pseudomonas aeruginosa C3719* | 401 |
| Q1I979 | Gamma proteobacteria | *Pseudomonas entomophila (strain L48)* | 456 |
| Q4KBX8 | Gamma proteobacteria | *Pseudomonas fluorescens (strain Pf-5/ATCC BAA-477)* | 395 |
| Q3KEB9 | Gamma proteobacteria | *Pseudomonas fluorescens (strain PfO-1)* | 447 |
| A4XUH1 | Gamma proteobacteria | *Pseudomonas mendocina (strain ymp)* | 401 |
| A4Y0F8 | Gamma proteobacteria | *Pseudomonas mendocina (strain ymp)* | 444 |
| A4XUJ0 | Gamma proteobacteria | *Pseudomonas mendocina (strain ymp)* | 453 |
| A5W577 | Gamma proteobacteria | *Pseudomonas putida (strain F1 / ATCC 700007)* | 450 |
| B0KJP7 | Gamma proteobacteria | *Pseudomonas putida (strain GB-1)* | 456 |
| Q88JU1 | Gamma proteobacteria | *Pseudomonas putida (strain KT2440)* | 450 |
| B1J9U1 | Gamma proteobacteria | *Pseudomonas putida (strain W619)* | 408 |
| B1JFP9 | Gamma proteobacteria | *Pseudomonas putida (strain W619)* | 456 |
| A4VM73 | Gamma proteobacteria | *Pseudomonas stutzeri (strain A1501)* | 406 |
| A4VNL6 | Gamma proteobacteria | *Pseudomonas stutzeri (strain A1501)* | 465 |
| Q1QCD2 | Gamma proteobacteria | *Psychrobacter cryohalolentis (strain K5)* | 402 |
| A1SX52 | Gamma proteobacteria | *Psychromonas ingrahamii (strain 37)* | 420 |
| A4BCW7 | Gamma proteobacteria | *Reinekea sp. MED297* | 392 |
| Q21DU0 | Gamma proteobacteria | *Saccharophagus degradans (strain 2-40 / ATCC 43961 / DSM 17024)* | 387 |
| A8R6M2 | Gamma proteobacteria | *Salmonella enterica subsp. enterica serovar Choleraesuis* | 401 |
| Q5QJF8 | Gamma proteobacteria | *Salmonella typhimurium* | 401 |
| A1S388 | Gamma proteobacteria | *Shewanella amazonensis (ATCC BAA-1098 / SB2B)* | 381 |
| A3D0Y1 | Gamma proteobacteria | *Shewanella baltica (strain OS155 / ATCC BAA-1091)* | 382 |
| A6WS33 | Gamma proteobacteria | *Shewanella baltica (strain OS185)* | 418 |
| A9L1I4 | Gamma proteobacteria | *Shewanella baltica (strain OS195)* | 418 |
| A5NE29 | Gamma proteobacteria | *Shewanella baltica OS223* | 418 |
| Q12JE0 | Gamma proteobacteria | *Shewanella denitrificans (strain OS217 / ATCC BAA-1090 / DSM 15013)* | 383 |
| Q087X4 | Gamma proteobacteria | *Shewanella frigidimarina (strain NCIMB 400)* | 383 |
| B0TTN8 | Gamma proteobacteria | *Shewanella halifaxensis (strain HAW-EB4)* | 404 |
| A3QB26 | Gamma proteobacteria | *Shewanella loihica (strain ATCC BAA-1088 / PV-4)* | 402 |
| Q8EI63 | Gamma proteobacteria | *Shewanella oneidensis* | 390 |
| A8H0J3 | Gamma proteobacteria | *Shewanella pealeana (strain ATCC 700345/ANG-SQ1)* | 395 |
| A4Y9Y3 | Gamma proteobacteria | *Shewanella putrefaciens (strain CN-32/ATCC BAA-453)* | 383 |
| A2UZT1 | Gamma proteobacteria | *Shewanella putrefaciens 200* | 383 |
| A8FQR9 | Gamma proteobacteria | *Shewanella sediminis (strain HAW-EB3)* | 378 |
| A0L0F5 | Gamma proteobacteria | *Shewanella sp. (strain ANA-3)* | 390 |
| A0L3E7 | Gamma proteobacteria | *Shewanella sp. (strain ANA-3)* | 455 |
| Q0HM14 | Gamma proteobacteria | *Shewanella sp. (strain MR-4)* | 390 |
| Q0HRS1 | Gamma proteobacteria | *Shewanella sp. (strain MR-7)* | 390 |
| A1RGE7 | Gamma proteobacteria | *Shewanella sp. (strain W3-18-1)* | 383 |
| B1KFR3 | Gamma proteobacteria | *Shewanella woodyi (strain ATCC 51908 / MS32)* | 386 |
| B4SIU1 | Gamma proteobacteria | *Stenotrophomonas maltophilia (strain R551-3)* | 400 |
| Q31EY2 | Gamma proteobacteria | *Thiomicrospira crunogena (strain XCL-2)* | 392 |
| Q1VF83 | Gamma proteobacteria | *Vibrio alginolyticus 12G01* | 379 |
| Q1ZVM0 | Gamma proteobacteria | *Vibrio angustum S14* | 383 |
| A8T6T5 | Gamma proteobacteria | *Vibrio campbellii AND4* | 379 |
| Q9KPM8 | Gamma proteobacteria | *Vibrio cholerae* | 380 |
| A2P6W6 | Gamma proteobacteria | *Vibrio cholerae 1587* | 380 |
| A1F2C8 | Gamma proteobacteria | *Vibrio cholerae 2740-80* | 380 |
| A6A9W8 | Gamma proteobacteria | *Vibrio cholerae 623-39* | 380 |
| A6XUR4 | Gamma proteobacteria | *Vibrio cholerae AM-19226* | 380 |
| A3GX95 | Gamma proteobacteria | *Vibrio cholerae B33* | 380 |
| A2PG56 | Gamma proteobacteria | *Vibrio cholerae MAK 757* | 380 |
| A3EDR1 | Gamma proteobacteria | *Vibrio cholerae MO10* | 380 |
| A6A3M8 | Gamma proteobacteria | *Vibrio cholerae MZO-2* | 380 |
| A2PQE0 | Gamma proteobacteria | *Vibrio cholerae MZO-3* | 380 |
| A3GLX1 | Gamma proteobacteria | *Vibrio cholerae NCTC 8457* | 380 |
| A6Y365 | Gamma proteobacteria | *Vibrio cholerae RC385* | 380 |
| A5F5U7 | Gamma proteobacteria | *Vibrio cholerae serotype O1 (strain ATCC 39541 / Ogawa 395 / O395)* | 380 |
| A1EKE9 | Gamma proteobacteria | *Vibrio cholerae V52* | 380 |
| Q5E7C8 | Gamma proteobacteria | *Vibrio fischeri (strain ATCC 700601 / ES114)* | 383 |
| B5FAR6 | Gamma proteobacteria | *Vibrio fischeri (strain MJ11)* | 383 |
| A7MUY9 | Gamma proteobacteria | *Vibrio harveyi (strain ATCC BAA-1116 / BB120)* | 390 |
| A6ARN4 | Gamma proteobacteria | *Vibrio harveyi HY01* | 390 |
| Q87M51 | Gamma proteobacteria | *Vibrio parahaemolyticus* | 379 |
| A6AZK3 | Gamma proteobacteria | *Vibrio parahaemolyticus AQ3810* | 379 |
| A6D204 | Gamma proteobacteria | *Vibrio shilonii AK1* | 403 |
| A7K353 | Gamma proteobacteria | *Vibrio sp. Ex25* | 379 |
| A3Y0V5 | Gamma proteobacteria | *Vibrio sp. MED222* | 382 |
| A3URT5 | Gamma proteobacteria | *Vibrio splendidus 12B01* | 382 |
| Q8DBQ1 | Gamma proteobacteria | *Vibrio vulnificus* | 383 |
| Q7MI68 | Gamma proteobacteria | *Vibrio vulnificus (strain YJ016)* | 383 |
| A5KZ42 | Gamma proteobacteria | *Vibrionales bacterium SWAT-3* | 382 |
| Q1IS27 | Acidobacteria | *Acidobacteria bacterium (strain Ellin345)* | 379 |
| A3HY30 | Bacteriodetes | *Algoriphagus sp. PR1* | 398 |
| Q11T46 | Bacteriodetes | *Cytophaga hutchinsonii (ATCC 33406 / NCIMB 9469)* | 385 |
| Q11PK4 | Bacteriodetes | *Cytophaga hutchinsonii (ATCC 33406 / NCIMB 9469)* | 400 |
| A8UEX8 | Bacteriodetes | *Flavobacteriales bacterium ALC-1* | 374 |
| A4APT3 | Bacteriodetes | *Flavobacteriales bacterium HTCC2170* | 373 |
| A5FBK1 | Bacteriodetes | *Flavobacterium johnsoniae (strain ATCC 17061 / DSM 2064 / UW101) (Cytophaga johnsonae)* | 376 |
| A5FFK5 | Bacteriodetes | *Flavobacterium johnsoniae (strain ATCC 17061 / DSM 2064 / UW101) (Cytophaga johnsonae)* | 376 |
| A1ZNZ4 | Bacteriodetes | *Microscilla marina ATCC 23134* | 400 |
| A6ECS6 | Bacteriodetes | *Pedobacter sp. BAL39* | 426 |
| A0H5R6 | Chloroflexi | *Chloroflexus aggregans DSM 9485* | 444 |
| A9WG50 | Chloroflexi | *Chloroflexus aurantiacus (strain ATCC 29366 / DSM 635 / J-10-fl)* | 394 |
| B2QPQ7 | Chloroflexi | *Chloroflexus sp. Y-400-fl* | 394 |
| A9AUV7 | Chloroflexi | *Herpetosiphon aurantiacus (ATCC 23779 / DSM 785)* | 388 |
| A7NLE1 | Chloroflexi | *Roseiflexus castenholzii (strain DSM 13941 / HLO8)* | 401 |
| A5UW23 | Chloroflexi | *Roseiflexus sp. (strain RS-1)* | 411 |
| B0C787 | Cyanobacteria | *Acaryochloris marina (strain MBIC 11017)* | 409 |
| Q8YXU9 | Cyanobacteria | *Anabaena sp. (strain PCC 7120)* | 402 |
| Q3M8M8 | Cyanobacteria | *Anabaena variabilis (strain ATCC 29413 / PCC 7937)* | 393 |
| Q4CB65 | Cyanobacteria | *Crocosphaera watsonii* | 389 |
| B1WXK0 | Cyanobacteria | *Cyanothece (strain ATCC 51142)* | 390 |
| B2EY51 | Cyanobacteria | *Cyanothece sp. PCC 7424* | 393 |
| B4CGN2 | Cyanobacteria | *Cyanothece sp. PCC 7425* | 404 |
| B4AZM5 | Cyanobacteria | *Cyanothece sp. PCC 7822* | 390 |
| B4BVE8 | Cyanobacteria | *Cyanothece sp. PCC 8802* | 387 |
| Q7NN49 | Cyanobacteria | *Gloeobacter violaceus* | 423 |
| B0JK87 | Cyanobacteria | *Microcystis aeruginosa (strain NIES-843)* | 385 |
| A8YG66 | Cyanobacteria | *Microcystis aeruginosa PCC 7806* | 385 |
| B2J7I3 | Cyanobacteria | *Nostoc punctiforme (strain ATCC 29133 / PCC 73102)* | 406 |
| Q0GPN9 | Cyanobacteria | *Prochlorococcus marinus (strain MIT 9301)* | 408 |
| A2C8R3 | Cyanobacteria | *Prochlorococcus marinus (strain MIT 9303)* | 394 |
| Q31BG2 | Cyanobacteria | *Prochlorococcus marinus (strain MIT 9312)* | 408 |
| Q0GPN8 | Cyanobacteria | *Prochlorococcus marinus (strain NATL1A)* | 412 |
| Q46KP3 | Cyanobacteria | *Prochlorococcus marinus (strain NATL2A)* | 412 |
| Q7V1Y4 | Cyanobacteria | *Prochlorococcus marinus subsp. pastoris (strain CCMP1378 / MED4)* | 408 |
| Q31R97 | Cyanobacteria | *Synechococcus elongatus (strain PCC 7942) (Anacystis nidulans R2)* | 383 |
| Q55027 | Cyanobacteria | *Synechococcus elongatus (strain PCC 7942) (Anacystis nidulans R2)* | 393 |
| Q5N305 | Cyanobacteria | *Synechococcus sp. (strain ATCC 27144 / PCC 6301 / SAUG 1402/1) (Anacystis nidulans)* | 383 |
| B1XLM4 | Cyanobacteria | *Synechococcus sp. (strain ATCC 27264 / PCC 7002 / PR-6) (Agmenellum quadruplicatum)* | 379 |
| Q3AKM4 | Cyanobacteria | *Synechococcus sp. (strain CC9605)* | 394 |
| Q2JM15 | Cyanobacteria | *Synechococcus sp. (strain JA-2-3B'a(2-13)) (Cyanobacteria bacterium Yellowstone B-Prime)* | 423 |
| B1U5W8 | Cyanobacteria | *Synechococcus sp. (strain PCC 8801 / RF-1) (Cyanothece PCC 8801)* | 387 |
| B1U211 | Cyanobacteria | *Synechococcus sp. (strain PCC 8801 / RF-1) (Cyanothece PCC 8801)* | 472 |
| A5GT28 | Cyanobacteria | *Synechococcus sp. (strain RCC307)* | 401 |
| A5GKZ8 | Cyanobacteria | *Synechococcus sp. (strain WH7803)* | 395 |
| A4CUR0 | Cyanobacteria | *Synechococcus sp. (strain WH7805)* | 395 |
| Q7U6L5 | Cyanobacteria | *Synechococcus sp. (strain WH8102)* | 394 |
| B4WGB1 | Cyanobacteria | *Synechococcus sp. PCC 7335* | 397 |
| A3Z701 | Cyanobacteria | *Synechococcus sp. RS9917* | 397 |
| P74550 | Cyanobacteria | *Synechocystis sp. (strain PCC 6803)* | 399 |
| Q6ZEU2 | Cyanobacteria | *Synechocystis sp. (strain PCC 6803)* | 412 |
| Q111F9 | Cyanobacteria | *Trichodesmium erythraeum (strain IMS101)* | 416 |
| B0SAX7 | Spirochaetes | *Leptospira biflexa serovar Patoc (strain Patoc 1/Ames)* | 390 |
| B0SIY4 | Spirochaetes | *Leptospira biflexa serovar Patoc (strain Patoc 1/ATCC 23582 / Paris)* | 390 |
| B2ENZ6 | Verrucomicrobia | *Bacterium Ellin514* | 435 |
| B4D986 | Verrucomicrobia | *Chthoniobacter flavus Ellin428* | 383 |
| A7CSJ7 | Verrucomicrobia | *Opitutaceae bacterium TAV2* | 401 |
| B1ZNT5 | Verrucomicrobia | *Opitutus terrae (strain DSM 11246 / PB90-1)* | 398 |
| A0AWW9 | Actinobacteria | *Arthrobacter sp. (strain FB24)* | 450 |
| A6YFR6 | Actinobacteria | *Arthrobacter sp. Chr15* | 452 |
| Q8FN03 | Actinobacteria | *Corynebacterium efficiens* | 377 |
| Q8NMW5 | Actinobacteria | *Corynebacterium glutamicum (Brevibacterium flavum)* | 376 |
| A4QGJ1 | Actinobacteria | *Corynebacterium glutamicum (strain R)* | 376 |
| Q2JCK7 | Actinobacteria | *Frankia sp. (strain CcI3)* | 398 |
| A6W599 | Actinobacteria | *Kineococcus radiotolerans (strain ATCC BAA-149 / DSM 14245 / SRS30216)* | 404 |
| A1SGG1 | Actinobacteria | *Nocardioides sp. (strain BAA-499 / JS614)* | 376 |
| A1SGW1 | Actinobacteria | *Nocardioides sp. (strain BAA-499 / JS614)* | 452 |
| Q0SC58 | Actinobacteria | *Rhodococcus sp. (strain RHA1)* | 467 |
| Q1ASM3 | Actinobacteria | *Rubrobacter xylanophilus (DSM 9941/NBRC 16129)* | 378 |
| Q1J261 | Deinococcus-Thermus | *Deinococcus geothermalis (strain DSM 11300)* | 387 |
| Q9RRS2 | Deinococcus-Thermus | *Deinococcus radiodurans* | 400 |
| A6TNK0 | Firmicutes | *Alkaliphilus metalliredigens (strain QYMF)* | 399 |
| A8MG88 | Firmicutes | *Alkaliphilus oremlandii (strain OhILAs) (Clostridium oremlandii (strain OhILAs))* | 399 |
| Q6HQU5 | Firmicutes | *Bacillus anthracis* | 393 |
| Q81X10 | Firmicutes | *Bacillus anthracis* | 393 |
| B1USR3 | Firmicutes | *Bacillus anthracis str. A0174* | 393 |
| B0Q5Z4 | Firmicutes | *Bacillus anthracis str. A0193* | 393 |
| B1F235 | Firmicutes | *Bacillus anthracis str. A0389* | 393 |
| B0QLE1 | Firmicutes | *Bacillus anthracis str. A0442* | 393 |
| B1GKW8 | Firmicutes | *Bacillus anthracis str. A0465* | 393 |
| B0AP14 | Firmicutes | *Bacillus anthracis str. A0488* | 393 |
| B3J879 | Firmicutes | *Bacillus anthracis Tsiankovskii-I* | 393 |
| Q9L4R8 | Firmicutes | *Bacillus cereus* | 393 |
| Q9XBH5 | Firmicutes | *Bacillus cereus* | 393 |
| Q72XQ8 | Firmicutes | *Bacillus cereus (strain ATCC 10987)* | 393 |
| Q815F2 | Firmicutes | *Bacillus cereus (strain ATCC 14579 / DSM 31)* | 393 |
| Q631E8 | Firmicutes | *Bacillus cereus (strain ZK / E33L)* | 393 |
| B3ZR23 | Firmicutes | *Bacillus cereus 03BB108* | 393 |
| B5UQ00 | Firmicutes | *Bacillus cereus AH1134* | 393 |
| Q4MIL0 | Firmicutes | *Bacillus cereus G9241* | 393 |
| B5V8N8 | Firmicutes | *Bacillus cereus H3081.97* | 393 |
| B3Z8S8 | Firmicutes | *Bacillus cereus NVH0597-99* | 393 |
| B3YQ47 | Firmicutes | *Bacillus cereus W* | 393 |
| Q5WKH8 | Firmicutes | *Bacillus clausii (strain KSM-K16)* | 391 |
| Q9KFB1 | Firmicutes | *Bacillus halodurans* | 397 |
| Q62NX4 | Firmicutes | *Bacillus licheniformis (strain DSM 13 / ATCC 14580)* | 397 |
| Q65DF4 | Firmicutes | *Bacillus licheniformis (strain DSM 13 / ATCC 14580)* | 398 |
| A3ID47 | Firmicutes | *Bacillus sp. B14905* | 400 |
| Q2BA20 | Firmicutes | *Bacillus sp. NRRL B-14911* | 405 |
| A6CJD2 | Firmicutes | *Bacillus sp. SG-1* | 394 |
| A0RKZ2 | Firmicutes | *Bacillus thuringiensis (strain Al Hakam)* | 393 |
| Q3EY30 | Firmicutes | *Bacillus thuringiensis serovar israelensis ATCC 35646* | 393 |
| Q6HB83 | Firmicutes | *Bacillus thuringiensis subsp. konkukian* | 393 |
| Q6HIU6 | Firmicutes | *Bacillus thuringiensis subsp. konkukian* | 397 |
| A9VR49 | Firmicutes | *Bacillus weihenstephanensis (strain KBAB4)* | 393 |
| B1I2K6 | Firmicutes | *Desulforudis audaxviator (strain MP104C)* | 409 |
| B1YLL8 | Firmicutes | *Exiguobacterium sibiricum (DSM 17290/JCM 13490 / 255-15)* | 385 |
| B2QAA3 | Firmicutes | *Exiguobacterium sp. AT1b* | 394 |
| Q5KZD3 | Firmicutes | *Geobacillus kaustophilus* | 400 |
| B3KIV5 | Firmicutes | *Geobacillus sp. Y412MC10* | 409 |
| B1HUD5 | Firmicutes | *Lysinibacillus sphaericus (strain C3-41)* | 398 |
| Q8ELF3 | Firmicutes | *Oceanobacillus iheyensis* | 400 |
| B1DLJ7 | Firmicutes | *Paenibacillus sp. JDR-2* | 390 |
| A9NGP5 | Tenericutes | *Acholeplasma laidlawii (strain PG-8A)* | 393 |
| Q5W3A9 | Uncultured bacterium | *Uncultured bacterium* | 401 |
| Q8RSK4 | Uncultured bacterium | *Uncultured bacterium* | 410 |
| Q4JMT1 | Uncultured bacterium | *Uncultured bacterium BAC17H8* | 459 |
| Q4PNF8 | Uncultured bacterium | *Uncultured marine bacterium 66A03* | 422 |
| Q18IC0 | Euryarchaeota | *Haloquadratum walsbyi (strain DSM 16790)* | 452 |
| Q58128 | Euryarchaeota | *Methanocaldococcus jannaschii* | 402 |
| A5YSN6 | Euryarchaeota | *Uncultured haloarchaeon* | 452 |
| XP_002183024.1 | Bacillariophyta | *Phaeodactylum_tricornutum_CCAP1055/1* | 493 |
| EDO96705.1 | Chlorophyta | *Chlamydomonas_reinhardtii* | 393 |
| A4S8V6 | Chlorophyta | *Ostreococcus lucimarinus (strain CCE9901)* | 476 |
| Q00TQ5 | Chlorophyta | *Ostreococcus tauri* | 492 |
| EDN08151.1 | Ascomycota | *Ajellomyces capsulatus_NAm1* | 538 |
| EAS33667.1 | Ascomycota | *Coccidioides immitisRS* | 535 |
| Q5B203 | Ascomycota | *Emericella nidulans* | 523 |
| Q5B7Y9 | Ascomycota | *Emericella nidulans* | 510 |
| EDJ94721.1 | Ascomycota | *Magnaporthe grisea 70-15* | 493 |
| EAW20015.1 | Ascomycota | *Neosartorya fischeri NRRL 181* | 524 |
| XP_961667.2 | Ascomycota | *Neurospora crassa OR74A* | 507 |
| Q8WZQ7 | Ascomycota | *Neurospora crassa* | 584 |
| XP_001911252.1 | Ascomycota | *Podospora anserina* | 505 |
| XP_001908554.1 | Ascomycota | *Podospora anserina* | 519 |
| EDU50222.1 | Ascomycota | *Pyrenophora tritici-repentis Pt-1C-BFP* | 536 |
| EED24596.1 | Ascomycota | *Talaromyces stipitatus ATCC 10500* | 475 |
| EED22782.1 | Ascomycota | *Talaromyces stipitatus ATCC10500* | 521 |
| EAU84631.1 | Basidiomycota | *Coprinopsis cinerea okayama7#130* | 531 |
| EAU84640.1 | Basidiomycota | *Coprinopsis cinerea_okayama7#130* | 532 |
| EDR01036.1 | Basidiomycota | *Laccaria bicolor S238N-H82* | 481 |
| EDR05068.1 | Basidiomycota | *Laccaria bicolor S238N-H82* | 468 |
| EAW13587.1 | Deuteromycota | *Aspergillus clavatus NRRL 1* | 525 |
| EDP50810.1 | Deuteromycota | *Aspergillus fumigatus A1163* | 382 |
| EAL84903.1 | Deuteromycota | *Aspergillus fumigatus Af293* | 491 |
| CAK43494.1 | Deuteromycota | *Aspergillus niger* | 483 |
| BAE62052.1 | Deuteromycota | *Aspergillus oryzae RIB40* | 532 |
| EAU39532.1 | Deuteromycota | *Aspergillus terreus NIH2624* | 495 |
| CAP93015.1 | Deuteromycota | *Penicillium chrysogenum Wisconsin 54-1255* | 523 |
| EEA23553.1 | Deuteromycota | *Penicillium marneffei ATCC 18224* | 527 |
| EEA20063.1 | Deuteromycota | *Penicillium marneffei ATCC 18224* | 514 |
