# Supplementary material for: High-level chromate resistance in Arthrobacter sp. strain FB24 requires previously uncharacterized accessory genes
Source: BMC Microbiol. 2009 Sep 16;9:199. doi: 10.1186/1471-2180-9-199 (PMC2751784; doi:10.1186/1471-2180-9-199)
Supplement: Additional file 4 — Supplemental Table S2. Recipe for vitamin solution added to mXBM. [file 1471-2180-9-199-S4.DOC]

**Supplementary Table S2: Vitamin Solution used for mXBM. All amounts are per liter solution. 10 ml of Vitamin solution added to 1 L of mXBM [1].**

| Vitamin | Milligrams (mg) |
| --- | --- |
| Biotin | 2 |
| Folic acid | 2 |
| Pyridoxine HCl | 10 |
| Riboflavin | 5 |
| Thiamine HCl | 5 |
| Nicotinic acid | 5 |
| Ca Pantothenate | 5 |
| Vitamin B12 | 0.1 |
| PABA (p-aminobenzoic acid) | 5 |

1. Jerke KH**: Physiological and Genetic Analysis of Plasmid-Mediated Metal Resistance i*n Arthrobact*er sp strain AK**-1. West Lafayette: Purdue University; 2006.
